# Supplementary material for: Acceptance and Commitment Therapy for Muscle Disease (ACTMus): protocol for a two-arm randomised controlled trial of a brief guided self-help ACT programme for improving quality of life in people with muscle diseases
Source: BMJ Open. 2018 Oct 3;8(10):e022083. doi: 10.1136/bmjopen-2018-022083 (PMC6194473; doi:10.1136/bmjopen-2018-022083)
Supplement: Author's manuscript [file bmjopen-2018-022083.draft_revisions.pdf]

BMJ Open is committed to open peer review. As part of this commitment we make the peer review history of every article we publish publicly available.

When an article is published we post the peer reviewers' comments and the authors' responses online. We also post the versions of the paper that were used during peer review. These are the versions that the peer review comments apply to.

The versions of the paper that follow are the versions that were submitted during the peer review process. They are not the versions of record or the final published versions. They should not be cited or distributed as the published version of this manuscript.

BMJ Open is an open access journal and the full, final, typeset and author-corrected version of record of the manuscript is available on our site with no access controls, subscription charges or pay-per-view fees (http://bmjopen.bmj.com).

If you have any questions on BMJ Open's open peer review process please email info.bmjopen@bmj.com

BMJ Open

Acceptance and Commitment Therapy for MuScle disease (ACTMuS): protocol for a two-arm randomised controlled trial of a brief guided self-help ACT programme for improving quality of life in people with muscle diseases

Journal:	*BMJ Open*
Manuscript ID	bmjopen-2018-022083
Article Type:	Protocol
Date Submitted by the Author:	09-Feb-2018

Complete List of Authors:	Rose, Michael ; King's College Hospital, Department of Neurology<br>Norton, Sam; King's College London, Institute of Psychiatry, Psychology and Neuroscience, Department of Psychology<br>Vari, Chiara; King's College London, Institute of Psychiatry, Psychology and Neuroscience, Department of Psychological Medicine<br>Edwards, Victoria; King's College London, Institute of Psychiatry, Psychology and Neuroscience, Department of Psychological Medicine<br>McCracken, Lance; King's College London, Institute of Psychiatry, Psychology and Neuroscience, Department of Psychology<br>Graham, Christopher; University of Leeds, Leeds Institute of Health Sciences<br>Radunovic, Aleksandar; Royal London Hospital, Barts and the London MND Centre<br>Chalder, Trudie; King's College London, Institute of Psychiatry, Psychology and Neuroscience, Department of Psychological Medicine

Keywords:	Muscle diseases, Quality of life, Acceptance and commitment therapy, Randomised control trial, Adult

SCHOLARONE™ Manuscripts

For peer review only

Acceptance and Commitment Therapy for MuScle disease (ACTMuS): protocol for a two-arm randomised controlled trial of a brief guided self-help ACT programme for improving quality of life in people with muscle diseases

Michael R. Rose (corresponding author), King's College Hospital, Department of Neurology, Denmark Hill, London, SE5 9RS

Email: m.r.rose@kcl.ac.uk

Telephone: 020 3299 8343

Michael R. Rose, King's College Hospital, Department of Neurology, London, UK

Sam Norton, King's College London, Institute of Psychiatry, Psychology and Neuroscience, Department of Psychology, London, UK

Chiara Vari, King's College London, Institute of Psychiatry, Psychology and Neuroscience, Department of Psychological Medicine, London, UK

Victoria Edwards, King's College London, Institute of Psychiatry, Psychology and Neuroscience, Department of Psychological Medicine, London, UK

Lance McCracken, King's College London, Institute of Psychiatry, Psychology and Neuroscience, Department of Psychology, London, UK

Christopher D. Graham, University of Leeds, Leeds Institute of Health Sciences, Leeds, UK

Aleksandar Radunovic, Royal London Hospital, Barts and the London MND Centre, London, UK,

Trudie Chalder, King's College London, Institute of Psychiatry, Psychology and Neuroscience, Department of Psychological Medicine, London, UK

Key words: Muscle diseases; quality of life; acceptance and commitment therapy; randomised control trial; adults

Word count: 5,129

For peer review only - http://bmjopen.bmj.com/site/about/guidelines.xhtml

ABSTRACT

Introduction: In adults, muscle disease (MD) is most often a chronic long term condition with no definitive cure. It causes wasting and weakness of the muscles resulting in a progressive decline in mobility, alongside other symptoms, and is typically associated with reduced quality of life (QoL). Previous research suggests that a psychological intervention, and in particular Acceptance and Commitment Therapy (ACT), may help improve QoL in MD. ACT is a newer type of cognitive behavioural treatment that aims to improve QoL by virtue of improvement in a process called psychological flexibility. The primary aim of this randomised control trial (RCT) is to evaluate the efficacy of a guided self-help ACT programme for improving QoL in people with MD. Main secondary outcomes are mood, symptom impact, work and social adjustment and function at 9-week follow-up.

Methods and analysis: ACTMuS is an assessor-blind, multicentre, two-armed, parallel-group RCT to assess the efficacy of ACT plus standard medical care (SMC) compared to SMC alone. Individuals with a diagnosis of one of four specific MDs, with a duration of at least 6 months, and with mild to moderate anxiety or depression (HADS score ≥ 8) will be recruited from UK-based MD clinics and MD patient support organisations. Participants will be randomised to either ACTplusSMC or SMC alone by an independent randomisation service. Participants will be followed up at 3, 6 and 9 weeks. Analysis will be intention to treat and will be conducted by the trial statistician who will be blinded to treatment allocation.

Ethics and dissemination: The study has received full ethical approval. Study results will be disseminated via peer-reviewed publications, conference presentations and journal articles. Data obtained from the trial will enable clinicians and health service providers to make informed decisions regarding the efficacy of ACT for improving QoL for patients with MD.

For peer review only

STRENGTHS AND LIMITATIONS OF THIS STUDY:

- ACTMuS is the first randomised controlled trial to test the efficacy of an ACT-informed intervention for MDs against standard medical care.
- To our knowledge this is the largest trial of a psychological intervention for MD, and the first that primarily targets QoL.
- In order to improve access and increase uptake, the intervention is brief and designed to be delivered remotely (via e-mail and telephone).
- Only a short follow-up period was included and consequently this study will not be able to investigate longer-term treatment effects.

For peer review only

For peer review only - http://bmjopen.bmj.com/site/about/guidelines.xhtml

INTRODUCTION

Background and rationale

Muscle diseases (MDs) are a group of primary disorders of the muscle, the majority of which are chronic and progressive. They affect approximately over 70,000 people (children and adults) in the UK (1). The diagnosis of a specific MD is based on several criteria including the pattern of the muscle weakness, the muscle biopsy appearances and in some cases genetic testing. Four MDs, namely limb girdle muscular dystrophy (LGMD), Becker's muscular dystrophy (BMD) (dystrophin deficiency), facioscapulohumeral muscular dystrophy (FSHD), and inclusion body myositis (IBM) comprise a significant proportion of those seen in an adult muscle clinic. They are characterised by progressive limb muscle wasting and weakness that cause difficulties with mobility and physical functioning. Some MDs also involve bulbar muscles causing dysphagia, respiratory muscles causing respiratory compromise, and cardic muscle causing cardiac symptoms. As well as weakness commonly reported symptoms include chronic pain, sleep disturbance and fatigue (2, 3). Unlike some MDs these four MDs do not have additional multi-system or central nervous system involvement. At present most MDs are without disease-modifying treatments or cures.

Typically people with MD report reduced quality of life (QoL) and may experience increased levels of anxiety and depression (4, 5). A systematic review of 26 studies highlighted that, compared to controls, people with MD had compromised QoL in all areas of functioning (5). The degree of physical disability is a determinant of QoL in MDs (5). Therefore, clinical treatment is often directed at maintaining physical functioning. For example, physiotherapy and occupational therapy are applied to maximise remaining muscle function and patients are monitored for any cardiac or respiratory problems that may need supportive treatment. Even so, such interventions may have ability to improve QoL to a degree.

However physical disability does not explain all the variance in QoL (4, 6, 7). Psychological variables such as illness beliefs (6, 8), coping methods (9-11) and psychological flexibility (12) have been shown to account for variance in QoL. These findings are in line with a biopsychosocial model (13) where QoL is theorised to depend upon the interaction of psychological factors with biological and social factors.

The relationship between QoL and psychosocial factors in MD provides a treatment opportunity. In the absence of a direct cure for MD, psychological interventions may offer a way to enhance QoL. Encouragingly, a recent small RCT of a traditional cognitive behavioural intervention for improving fatigue in FSHD showed a beneficial effect on fatigue and QoL (14).

Acceptance and Commitment Therapy (ACT)

Dependent on treatment aims several types of psychological interventions may be helpful for people with MD (15). ACT is a newer form of cognitive-behavioural approach that focuses on improving a process called psychological flexibility (16). Psychological flexibility is "[…] the capacity to persist or to change behaviour in a way that includes conscious and open contact with thoughts and feelings, appreciates what the situation affords, and serves one's goals and values" (17). In observational studies psychological flexibility has been shown to be predictive of positive outcomes (QoL, mood) and better functioning across a range of challenging contexts, such as living with chronic illness and chronic pain. A 4-month prospective observational study with a group of people with MD observed that psychological flexibility was predictive of change in life satisfaction and anxiety (18).

Given its focus on improving psychological flexibility, ACT may prove beneficial for those with MD. In ACT a number of treatment techniques are used to improve psychological flexibility including therapist interaction, mindfulness and functional analysis. These aim to: a) help individuals become more aware of thoughts and feelings and their relationship to, in this case, their MD; b) broaden the range of behaviours (often to include open and willing responses) that can be applied when in the presence of difficult thoughts and feelings; c) help them to flexibly choose those behaviours that help them to make progress on their over-arching goals and values.

Several recent meta-analyses have suggested that ACT is an efficacious treatment for improving mood and QoL in mental health conditions (19, 20) and chronic pain (21). In chronic diseases there is a growing evidence base (12), with encouraging results for trials with cancer (22), diabetes (23), and epilepsy (24)

For peer review only - http://bmjopen.bmj.com/site/about/guidelines.xhtml

populations. However, to date, most studies of ACT for chronic diseases have been of a preliminary nature (i.e. small, uncontrolled, and/or lower quality (12). Thus, adequately-powered, rigorous evaluation of ACT for improving QoL in chronic diseases will be a helpful addition to the literature.

The selection of a guided self-help approach

The one comprehensive trial of a psychological intervention in MD (14) tested the efficacy of a 16 session traditional cognitive behavioural therapy for improving fatigue. While results were encouraging, both on fatigue and secondary outcomes such as QoL and mood, for several reasons (including the time spent travelling) uptake was low, and a proportion (24%) struggled to achieve even a minimal level of adherence to the intervention. This suggests that issues, perhaps mobility difficulties and treatment length, may hinder face-to-face treatment options for many with MD. A further problem with the development and implementation of any new treatment concerns the resource limitations common to many health service providers, including the NHS in the UK.

A practical solution to this problem could be the implementation of an intervention delivered remotely through online and computerised self-guided resources aided by telephone or video calls. Such methods of delivery are increasingly feasible given that the majority of UK households now have web access. Also recent systematic reviews suggest that promising results can be obtained from web-based interventions for adults with chronic illnesses and depression (25-27). Web based interventions not only remove the burden of travel, but also allows the completion of material at a convenient place, time, and pace. Remotely delivered interventions can be a cost-effective approach for providing treatment increasing its chances of wider implementation.

Given the potential benefits of using ACT within an MD population, and taking into account potential barriers to participation, we developed for this population a brief self-guided intervention that can be supported remotely via e-mail and telephone contact with a therapist. We assessed this in a preliminary multiple-baseline case series with seven participants (28). The results of this case series suggested that such an approach was practicable and acceptable and that mood and QoL might respond to this treatment. Given this promising evaluation we plan to conduct a randomised controlled trial, to evaluate the efficacy of this intervention for improving QoL in people living with MD.

This protocol describes a multicentre, two-armed, parallel-group RCT to assess the efficacy of self-guided ACT in improving QoL in individuals with MDs. A total of 154 people referred from MD clinics, Muscular Dystrophy UK and specific MD registries will be randomly allocated to receive either self-guided ACT plus standard medical care (SMC) or SMC alone. We hypothesise that the group who receive self-guided ACT plus SMC will report an improved QoL at the primary end point of 9 weeks follow-up compared with those receiving SMC alone.

Objectives

Primary objective:

1) The primary objective is to evaluate the efficacy of ACT plus SMC on QoL compared to SMC alone at 9-weeks.

Secondary objectives:

1) To evaluate the impact of ACT plus SMC compared to SMC alone on secondary outcomes: mood, symptom impact domains, work and social adjustment and function at 9-week follow-up.
2) To assess the processes that may be responsible for mediating the treatment effect.
3) To evaluate participants' views of the intervention and factors that act as facilitators and barriers to participation via a nested qualitative study.

Trial Design

ACTMuS is an assessor-blind, multicentre, two-armed, parallel-group randomised controlled trial (RCT).

For peer review only - http://bmjopen.bmj.com/site/about/guidelines.xhtml

METHOD: PARTICIPANTS, INTERVENTIONS AND OUTCOMES

Setting

A total of 154 participants will be recruited from UK National Health Service MD clinics, Muscular Dystrophy UK (MD-UK) and 3 disease-specific registries (FSHD, dysferlin, IBM). After obtaining consent, the baseline assessments will take place in MD clinics at King's College Hospital (NHS Foundation Hospital Trust), The Royal London Hospital (Barts Health NHS Trust) and University Hospital Southampton (NHS Foundation Trust). The remainder of the study including treatment will be conducted remotely via email, telephone or video calls.

Eligibility criteria

Inclusion criteria

1) Adults (aged 18 years and over) diagnosed with one of the following MDs that has been present for greater than 6 months:
    a. One of the limb girdle muscular dystrophies (LGMD) genetically or biopsy proven,
    b. Becker' muscular dystrophy (BMD) (dystrophin deficiency) with biopsy or genetic diagnosis,
    c. Facioscapulohumeral muscular dystrophy (FSHD) diagnosed clinically with specific genetic abnormality in the subject or their family,
    d. Inclusion body myositis (IBM) clinic-pathologically defined, clinically defined or probable IBM based on European Neuromuscular Centre ENMC research diagnostic criteria 2013 (29).
2) Potential participants must have access to the Internet and a computer to receive the study materials
3) HADS score of ≥ 8 for depression or ≥8 for anxiety.

Exclusion criteria

1) Unstable complications of MDs including neuromuscular respiratory weakness or cardiomyopathy.
2) Major active co-morbidities unrelated to MD (such as arthritis, respiratory disease, cardiovascular disease).
3) Current diagnosis of an active major mental health disorder likely to interfere with participation
4) Current or recent participation in other treatment intervention studies (< 4 weeks after completion).
5) Currently receiving psychological support or psychotherapy.
6) Inability to read English questionnaires
7) Cognitive impairment that would prevent comprehension of ACT modules and questionnaires (as assessed by the Montreal Cognitive Assessment 5-minute protocol, (30)).

INTERVENTIONS

Acceptance and Commitment Therapy

The intervention is a remotely supported, guided self-help ACT programme (developed mainly by CG and LM, informed by other sources (16, 22, 31). It consists of four modules and corresponding audio files supported by five telephone support sessions with the trial therapist (Table 1). The first three modules are expected to take the participant 1 hour 30 minutes each and the final module 45 minutes to complete. This includes time to read the modules, listen to the audio files and work through tasks and exercises. During the first call, prior to receiving the first module (either by telephone or video call), the therapist will provide a 15-minute introduction then email the first module. The subsequent three phone calls will each last 30-minutes and will be followed by the next module. The telephone sessions provide participants with a chance to discuss the modules they have read, including their experiences of the exercises, and receive help with difficulties encountered. A 15-minute phone call will follow the final module to conclude the treatment.

For peer review only - http://bmjopen.bmj.com/site/about/guidelines.xhtml

Module 1: Mindfulness and unhooking	• Normalising difficult thoughts and feelings, given the context of MD, and associated struggles.<br>• Discussion of consequences from trying to get rid of unwanted thoughts and feelings and opportunities for alternative responses.<br>• Introducing other ways to interact with unwanted thoughts and feelings:<br>1. Mindfulness or learning to be present in the moment and experience thoughts and feelings in a non-judgemental way.<br>2. Unhooking from thoughts or learning ways to step-back from thoughts that often restrict actions or lead to avoidance.<br>• Homework 1: Daily diary of mindfulness practice.<br>• Homework 2: Identifying where one struggles and experimenting with other responses.
Module 2: Follow your values	• Identifying values and ways to pursue them with MD. Exploring the link between difficult thoughts and feelings and one's goals and values.<br>• Homework 1: Continuation of daily mindfulness practice<br>• Homework 2: Setting goals in the context of values, experimenting with taking the 'smallest possible step' which is consistent with ones values, noticing the thoughts and feelings that occur in the context of values activity.
Module 3: Take an observer perspective	• Experimenting with taking an observer perspective on one's experiences.<br>• Considering labels one attaches to oneself (especially given the context of MD). Noticing a choice over buying into labels, and the impact of labels over one's behaviour.<br>• Introducing a more flexible approach to mindfulness<br>• Homework 1: Continuation of daily mindfulness practice with addition of flexible attention and noticing the observer self<br>• Homework 2: Continuation of valued activities
Module 4: Recap, reflection and suggestions for staying committed	• Review of homework tasks and skills learnt.<br>• Self-identification of effective and ineffective behaviour patterns.<br>• Goal planning and normalisation of set-backs with a compassionate approach to getting back on track.

Table 1 Summary of ACT modules

The Clinical Psychologist delivering the ACT support sessions will attend a three-day training session prior to the start of recruitment. The first day will cover the rationale behind the study and insights into MD diagnosis, symptoms and impact on QoL (led by MR, a Consultant Neurologist). Details of the study protocol will be reviewed as well as the role of the therapist, standard operating procedures, recording and storage of the support sessions and confidential information, deviations, dropouts and adverse event procedures (led by TC, Professor of Cognitive Behaviour Therapy). The ACT training including presentations, exercises and role-plays will last 2 days. This will be led by LM, a Clinical Psychologist and expert in ACT, assisted by CG, an ACT-trained Clinical Psychologist. Obstacles to patient engagement with ACT as well as the difficulties of delivering the sessions remotely and within a brief time frame will be discussed. The therapist will attend monthly supervision meetings with a clinical supervisor (LM) to develop their skills and to ensure therapy fidelity and adherence to trial protocol. The therapist's treatment competence will be periodically assessed with an ACT-fidelity scale. This will inform monthly supervision sessions between therapist and clinical supervisor throughout the trial.

Treatment fidelity

All ACT support sessions will be audio-recorded to assess treatment fidelity. At the end of the trial a random sample of recordings will be analysed for overall fidelity by two independent researchers. A fidelity measure is currently being developed by CG and LM. This comprises a basic therapeutic competence scale and an ACT-specific fidelity scale (ACT-FS) informed by guidelines provided by Plumb and Vilardaga (32).

For peer review only - http://bmjopen.bmj.com/site/about/guidelines.xhtml

Standard Medical Care (SMC)

All participants will receive SMC, which involves the continuation of any current medical practices (without any psychological therapy). As part of SMC, physiotherapists at the clinics provide personalised assessments and where appropriate additional, standardised information regarding the patient's MD, suitable registries and patient support organisations.

Baseline measures

Participants will complete baseline questionnaires (see Table 2) at the MD clinics with the research assistant (RA) and objective assessments with a physiotherapist. Participants will also complete a participant details form to provide demographic data as follows: age, gender, ethnicity, occupation status, education level, marital status, living arrangements and dependants. Clinical information will also be collected, including age of onset and duration of MD, additional diagnoses, experience of psychological therapies as well as prior treatment of anxiety or depression. Adverse events will be recorded at each assessment point of the trial.

The objective assessments conducted by physiotherapists at baseline are as follows:

- Adult Ambulatory Neuromuscular Assessment (ANA) is an adult version of the North Star Ambulatory Assessment (33) measures motor function. Items are scored from 0-2, with an overall possible range of scores from 0-34. The higher the score, the better the patient can function. An additional item of the ANA that shall be used is the 6-minute timed walk test (6MTWT) which measures mobility by counting the distance walked within a 6-minute time-frame along with pauses or stops before the time is complete. The higher the score the greater the function of the patient.

- Manual Muscle Strength Testing (MMST; aka Manual Muscle Testing, MMT) measures the strength of 12 different muscles on both right and left from 0-5 (no contraction palpable – normal strength). There is a total possible range of scores from 0- 120, with higher scores representing levels of strength closest to normal levels (34).

Outcome measures

The RA blind to participant allocation will be responsible for ensuring outcome measures are completed at 3, 6 and 9 weeks by all participants. The link to the measures will be sent to participants via email. If participants do not complete the questionnaires by the due date they will be reminded by the RA. All time points will be taken into account during analysis but the primary efficacy end-point is the 9-week follow-up.

Primary outcome:

- The Individualised Neuromuscular Quality of Life Questionnaire (INQoL) – life area domains (section 2) is the primary outcome. The INQoL is a 10-domain, validated MD specific questionnaire to measure QoL (35). We will exclude the one domain that addresses the symptom of mytonia as none of the MDs being recruited has this symptom. This will leave 9 domains for the purpose of this study. The primary outcome of this study constitutes section 2 of the INQOL which assesses 5 life area domains (activities, independence, social functioning, emotional functioning & body image). Participants respond using a 7-point Likert scale, with higher scores indicating greater symptom impact. The primary outcome measure for this study will therefore be the change in the overall QoL score, derived from these five life domain scores, from baseline up to 9-weeks.

For peer review only - http://bmjopen.bmj.com/site/about/guidelines.xhtml

Secondary outcome measures:

- Individualised Neuromuscular Quality of Life Questionnaire (INQoL) symptom impact domains. This refers to categories within Section 1 of the INQoL (weakness, pain, tiredness) and follows the same format as described above.

- Work and Social Adjustment Scale (WSAS) is a measure of impairment at work, home and social functioning (36). The total score comprises the sum of responses to five questions, each of which is on a nine-point Likert scale. The possible range of scores is 0-40; with higher scores indicating more greatly impaired daily functioning.

- Hospital Anxiety and Depression Scale (HADS) is a self-report questionnaire to measure mood (37). It consists of fourteen items, relating to depression (7 items) and anxiety (7 items). . Higher scores indicate either greater anxiety or depression. Each of the items are rated from 0-3 thus the possible range of scores for each is 0-21, with a cut-off of 8 showing good sensitivity and specificity for possible cases (38).

- Stanford Health Assessment Questionnaire Disability Index (HAQ-DI) is a measure of functional impairment and level of disability (39).There are different ways to score the HAQ-DI. As with previous studies with MD populations (18) the alternative scoring method shall be used which focuses on measuring disability not adaption by not incorporating use of aids and devices. The measure includes eight activity domains (dressing, arising, eating, walking, hygiene, reach, grip, and activities). There are 20 questions in total, each scored from 0-3. All the domains are summed for a total score with a higher score representing a greater impairment in ability to function in daily life. As additional measure of functional impairment, not treated as an efficacy outcome in the analysis, the IBM Functional Rating Scale (40) will be completed only by those participants with a diagnosis of Inclusion Body Myositis. This is a 10-point functional rating scale, with higher scores depicting greater functional impairment.

- Patient Global Impression of Change scale (PGIC) is a single item measure of a patient's impression of change over the study (41). Participants rate their impression of change on a scale of 1–7 as follows: 'very much better', 'much better', 'a little better', 'about the same', 'a little worse', 'much worse' and 'very much worse'.

- Rating of satisfaction is a single item measure of a patient's satisfaction with the outcome of the trial. Participants rate their satisfaction on a scale of 1–7 as follows: 'very satisfied', 'moderately satisfied', 'slightly satisfied', 'neither', 'slightly dissatisfied', 'moderately dissatisfied' and 'very dissatisfied'.

Putative Mediators and process variables:

- Acceptance and Action Questionnaire (AAQ-II) (42) measures psychological flexibility, including experiential avoidance and psychological inflexibility. Each of the seven items are rated on a scale of 1-7, with higher scores representing greater experiential avoidance and immobility.

- Mindfulness Attention Awareness Scale (MAAS) measures dispositional open awareness of and attention to the present moment (43). There are 15 items, each scored from 1-6. A mean of the 15 items is computed; with higher scores reflect higher levels of dispositional mindfulness.

- Committed Action Scale (CAQ) measures committed action i.e. goal-directed, flexible persistence (44). Respondents rate the extent to which the 8 items apply to them from 0-6 with a greater total score representing a greater general propensity to persist in goal-directed behaviour

For peer review only

For peer review only - http://bmjopen.bmj.com/site/about/guidelines.xhtml

Qualitative component

A nested qualitative study will explore patients' experience of the ACT intervention. This will help identify factors that facilitate or impede adherence and acceptability of the treatment, and perhaps give insight into treatment mechanisms. Semi-structured interviews will be conducted post-intervention with approximately 15 participants (purposively sampled to encompass a mix of gender, ages and symptom severity). Interviewers will use topic guides with open-ended questions and prompts to elicit participants' accounts of their experiences. The interviews will be transcribed verbatim. Analysis will commence after the completion of the first interview in an iterative process to allow exploration of early insights in later interviews. Standard thematic analysis techniques will be applied (45), for example, coding the data and identifying themes that capture key concepts and processes. The results will supplement quantitative results and inform any future implementation of the intervention.

For peer review only

For peer review only - http://bmjopen.bmj.com/site/about/guidelines.xhtml

Participant timeline

Information is provided in the consort diagram (Figure 1) and the screening and data schedule of assessments (Table 2). The RA will contact potentially interested participants to ensure they are fully informed and eligible to take part in the trial. If this is the case, they will be asked to sign a consent form, enrolled onto the study, and provided with a unique identification number. The baseline assessments will be in person at one of the MD clinics with the RA and physiotherapist. Following baseline, the participant will be randomised and informed by the trial coordinator as to whether they will receive ACT plus SMC or SMC alone. The participant will complete follow-up measures at 3, 6 and 9 weeks online via an email link.

Table 2 Screening and data collection across the trial: summary of key trial processes

Process	*Screening*	*Baseline*	*3 week*	*6 week*	*9 week*	*Ongoing or during trial*	*Ref*
Eligibility	X						-
Medical confirmation	X						-
HADs	X	X	X	X	X		37
MoCA	X						28
Participant details form		X					-
INQoL		X	X	X	X		33
WSAS		X	X	X	X		34
HAQ-DI		X	X	X	X		37
*IBM**		X	X	X	X		38
AAQ-II		X	X	X	X		40
MASS		X	X	X	X		41
CAQ		X	X	X	X		42
Health Events		X	X	X	X		-
ANA		X					31
6MTWT		X					31
MMST		X					32
PGIC			X	X	X		39
Satisfaction rating			X	X	X		-
Safety events (e.g. AE, SAEs)						X	-
Drop-out / withdrawal						X	-
Therapist ratings						X	-

For peer review only - http://bmjopen.bmj.com/site/about/guidelines.xhtml

KEY

**HADS**	Hospital Anxiety and Depression Scale
**MOCA**	Montreal Cognitive Assessment 5-minute protocol
**INQOL**	Individualised Neuromuscular Quality of Life Questionnaire
**WSAS**	Work and Social Adjustment Scale
**HAQ-DI**	Stanford Health Assessment Questionnaire Disability Index
**IBM**	IBM Functional Rating Scale
**AAQ-II**	Acceptance and Action Questionnaire
**MASS**	Mindfulness Attention Awareness Scale
**CAQ**	Committed Action Questionnaire
**ANA**	Adult Ambulatory Neuromuscular Assessment
**6MTWT**	6-minute timed walk test
**MMST**	Manual Muscle Strength Testing
**PGIC**	Patient Global Impression of Change scale

Sample size

154 patients will be recruited in total, 77 per trial arm. This sample size will provide adequate power to detect a standardised mean difference of 0.5 (medium magnitude) in the primary outcome between the treatment and control groups at the end of intervention assessment (5% significance, 80% power), assuming attrition of 20%. Using data from Vincent et al., (35) this equates to between an 11.5 and 15.8 point difference on each of the INQOL domains. While no information is currently available regarding clinically important differences for the INQOL, an effect size of this magnitude typically relates to a clinically important difference for health related quality of life instruments (46).

Recruitment

Participants will be recruited from MD clinics, Muscular Dystrophy UK and MD registries. In MD clinics, consultants and physiotherapists will identify potentially eligible participants who will either be given an information pack consisting of an invitation letter, information sheet and reply slip to state their interest. MD UK and MD registries will advertise the study and provide invitation packs. When someone states their interest, the RA will call them to provide further information, answer any questions and conduct screening. If they are eligible for the study (including medical confirmation from their health professional) then the participant will sign a consent form before being enrolled into the study.

METHODS: ASSIGNMENT OF INTERVENTIONS

Allocation

Randomisation will be conducted by an independent randomisation service at the UK Clinical Research Collaboration registered at the King's Clinical Trials Unit. Randomisation will be at the level of the individual, using block randomisation with randomly varying block sizes stratified by recruiting centre. Allocation confirmation will be generated automatically and sent to unblinded research members, including the trial co-ordinator and clinical psychologist.

For peer review only - http://bmjopen.bmj.com/site/about/guidelines.xhtml

ALLOCATION CONCEALMENT

A position of equipoise will be maintained but blinding of the patients and therapist to the treatment condition will not be possible. The RA contacting participants and inputting data will be blind to group allocation to reduce the risk of introducing bias. To protect against unblinding, all follow-up questionnaires will be completed by participants on Bristol Online Survey (BOS) which is specifically designed for academic research and fully compliant with UK data protection law. All questionnaires will be identical for both arms and designed not to reveal treatment allocation. The trial statistician will be blind to treatment group when conducting the main data analysis.

METHODS: DATA COLLECTION, MANAGEMENT AND ANALYSIS

Data collection Methods

Baseline data will be collected prior to randomisation and consist of self-report questionnaires and objective assessments with the physiotherapist. Follow-up data (3, 6 & 9 weeks) include self-report questionnaires that will be completed remotely. At each follow-up point participants will be emailed a link to BOS along with their unique identification number. If participants are unwilling or unable to complete all the questionnaires they will be given the option of going through them on the phone or a reduced questionnaires pack with the primary outcomes (INQoL).

Data Management

All trial data will be stored in line with the Data Protection Act 1998 and in compliance with Good Clinical Practice and King's College London data management procedures. Any identifying data will be anonymised through use of unique identification numbers separated from identifying data. Data will be entered onto password-protected trial databases either on a shared hard-drive accessible only to assigned team members or in a locked cabinet in a room only accessible to staff with designated access cards. All source documents will be retained for a period of 10 years following the end of the study.

Statistical methods

Statistical analyses of the primary and secondary outcomes will be conducted after the database has been locked, with no interim analyses. All analyses will adopt the intention to treat principle (ITT). The main statistical analyses will estimate the difference in mean outcomes between patients randomised to ACT plus SMC and SMC alone at the primary endpoint of 9 weeks and at the two other post-randomisation time points. Group difference estimates, associated confidence intervals and standardised effect sizes will be reported. Significance testing will be performed where the significance level will be 5% (2-sided). Sensitivity analyses will be used to assess the robustness of conclusions to missing outcome data using a pattern mixture model approach and to departures from randomised treatment in a per protocol analysis excluding those not receiving. Loss to follow-up, departures from randomised treatment and the prevalence of serious adverse events will be reported.

The primary outcome is the INQoL quality of life (QoL) score. Quality of life at all three post-randomisation time points (3, 6 and 9 weeks after randomisation) will be modelled simultaneously using a linear mixed-effects model. A random intercept will account for the non-independence of observations across individuals. Trial arm and indicator coded time will be included as covariates along with arm by time interaction terms to allow the treatment effect to vary at each time point. Also, included in the model will be the baseline level of the QoL score and a stratification indicator variable for centre. Missing data at follow up assessments is allowed where at least one observation per participant is available under the missing at random assumption.

Secondary patient outcomes (3, 6 and 9 weeks after randomisation) relating to symptom severity and functioning (WSAS; HAQ-DI), distress (HADS), impression of change (PGIC), and putative mediators of the intervention (CAQ; AAQ-II; MAAS) will be analysed using linear mixed-effects models. Random effects for the intercept and time will be included in the model.

For peer review only - http://bmjopen.bmj.com/site/about/guidelines.xhtml

METHODS: MONITORING

Data monitoring

The study will be monitored and audited in accordance with King's College Hospital NHS Foundation Trust and King's College London procedures. All trial related documentation will be made available on request to the sponsors, HRA/ REC and other licencing bodies. Monitoring of this study to ensure compliance with Good Clinical Practice and scientific integrity will be managed by the study team and reviewed by an independent Trial Steering Committee (TSC) & Data Monitoring & Ethics Committee (DMEC).

Harms/ Assessment of safety

Adverse events will be recorded at baseline, 3, 6 and 9 weeks. We define adverse events as 'any clinical change, disease or disorder experienced by the participant during their participation in the trial, whether or not considered related to the use of treatments being studied in the trial' (47). Adverse events will also include any other events that might have affected the health status of a participating patient (e.g. increased work stress).

The team will also monitor serious adverse events (SAEs), serious adverse reactions (SAR), suspected unexpected serious adverse reactions (SUSAR) and active withdrawals from treatment. A SAE constitutes any adverse event or reaction that: results in death or a new persistent or new significant disability or incapacity, is life threatening or requires hospitalisation, any new episode of deliberate self-harm or any other important medical condition which may jeopardise the participant and require medical or surgical intervention to prevent one of the outcomes listed above.

A serious adverse reaction (SAR) can be defined as a SAE as a reaction to the intervention. A SUSAR is any serious adverse reaction that is suspected to be caused by the intervention but was not expected. All SAEs, SARs and SUSARs will be reported immediately by the Chief Investigator to the study Sponsor and Research Ethics Committee. The TSC-DMEC will be responsible for the independent investigation of SAEs.

ETHICS AND DISSEMINATION

Research ethics approval

The trial is subject to Health Research Authority (HRA) approval that brings together the assessment of governance and legal compliance with the independent Research Ethics Committee (REC) approval. The trial has ethical approval by London - Camberwell St Giles REC (16/LO/0609). Site-specific confirmation of capacity and capability has been given by local research and development departments. The study will comply with the principles of the Declaration of Helsinki (1996) (48), the International Conference for Harmonisation of Good Clinical Practice (ICH GCP; (49)) guidelines and the Research Governance Framework for Health and Social Care 2nd Edition (2005). The trial is sponsored by King's College Hospital NHS Foundation Trust (lead) and King's College London and has been registered onto a publicly accessible database (Registration number: NCT02810028).

Consent

Inclusion in the trial is appropriate only if potential participants are aged 18 or older and are able to give written informed consent. All potentially eligible participants will be fully informed about the study procedures by a participant information sheet that states participation is voluntary and that they are free to withdraw from the study at any time without giving a reason and without their care being affected.

Declaration of interests

The authors declare that they have no competing interests

For peer review only - http://bmjopen.bmj.com/site/about/guidelines.xhtml

Dissemination

Summaries of findings will be offered to MD charities, registries, and support groups as well as to the wider public. The results of the trial will be communicated to participants via a newsletter. Any results from the trial will enable informed decisions regarding the provision of care for patients with MD.

CONCLUSION

Experiences of psychological distress and reduced QoL are common in people with MD. A brief, guided self-help ACT intervention with telephone support sessions has been designed for remote delivery, offering a potential solution to mobility issues inherent with MD and under-resourced healthcare services. This is the first study to evaluate whether ACT can help improve QoL for individuals with MD. It will also be one of the largest randomised controlled trials of ACT for improving outcomes in long-term conditions. If the intervention proves to be efficacious, this treatment delivery could facilitate participation for those individuals whose impaired mobility prevents travelling to face-to-face sessions. As the main barrier to the adoption of any psychological intervention is the paucity of psychological services, the ACT intervention has been designed to be a self-guided programme requiring minimal support from highly qualified professionals.

Abbreviations: ACT: Acceptance and Commitment Therapy, MD: Muscle Disease, SMC: Standard Medical Care QoL: Quality of Life, HRA: Health Research Authority, REC: Research Ethics Committee, AE: Adverse Event, SAE: Serious Adverse Event, SAR: Serious Adverse Reaction, SUSAR: Suspected Unexpected Serious Adverse Reaction, TSC-DMEC: Trial Steering Committee / Data Monitoring & Ethics Committee, PPI: patient and public involvement.

Acknowledgements: We are grateful to Daniel Thomas (PPI representative) and MD-UK representatives who commented on several drafts of the study materials at different stages. We are also grateful to our physiotherapy collaborators (Ms Joanna Reffin, Ms Emily Jay, Ms Sunitha Narayan, Ms Jade Donnelly, Mr Leslie Richards, Ms Kelly Orr).

Contributorship Statement: MR, CG, SN, LM, AR and TC obtained funding for this study. MR, SN, CV, VE, LM, CG, AR and TC contributed to the study protocol. MR and TC are responsible for overall supervision. All authors read, contributed towards and approved of the final manuscript.

Competing interests: None disclosed

Funding: National Institute for Health Research (NIHR) Research for Patient Benefit grant (Reference number: PB-PG-061331085). Muscular Dystrophy UK have been partners from the commencement of the study and funded the trial therapist.

The trial is sponsored by King's College Hospital NHS Foundation Trust and co-sponsored by King's College London.

For peer review only - http://bmjopen.bmj.com/site/about/guidelines.xhtml

References

1. Muscular Dystrophy Campaign. Muscle Disease: the Impact – Incidence and Prevalence of Neuromuscular Conditions in the UK. 2010. Published http://www.musculardystrophyuk.org/information-for-professionals/nhs-commissioners/resources-for-nhs-commissioners/neuromuscular-condition-prevalence/.

2. Della Marca G, Frusciante R, Vollono C, Iannaccone E, Dittoni S, Losurdo A, et al. Pain and the Alpha-Sleep Anomaly: A Mechanism of Sleep Disruption in Facioscapulohumeral Muscular Dystrophy. Pain Medicine. 2013;14(4):487-97.

3. Merrison A, Hanna M. Muscle disease. Practical neurology. 2009;9(1):54-65.

4. Burns TM, Graham CD, Rose MR, Simmons Z. Quality of life and measures of quality of life in patients with neuromuscular disorders. Muscle Nerve. 2012;46(1):9-25.

5. Graham CD, Rose MR, Grunfeld EA, Kyle SD, Weinman J. A systematic review of quality of life in adults with muscle disease. J Neurol. 2011;258(9):1581-92.

6. Graham CD, Weinman J, Sadjadi R, Chalder T, Petty R, Hanna MG, et al. A multicentre postal survey investigating the contribution of illness perceptions, coping and optimism to quality of life and mood in adults with muscle disease. Clin Rehabil. 2014 May;28(5):508-19.

7. Sadjadi R, Rose MR, Muscle Study Group. What determines quality of life in inclusion body myositis? J Neurol Neurosurg Psychiatry. 2010 Oct;81(10):1164-6.

8. Rose MR, Sadjadi R, Weinman J, Akhtar T, Pandya S, Kissel JT, et al. Role of disease severity, illness perceptions, and mood on quality of life in muscle disease. Muscle Nerve. 2012;46(3):351-9.

9. Nätterlund B, Gunnarsson L, Ahlström G. Disability, coping and quality of life in individuals with muscular dystrophy: a prospective study over five years. Disabil Rehabil. 2000;22(17):776-85.

10. Ahlstrom G, Sjoden P. Coping with illness-related problems and quality of life in adult individuals with muscular dystrophy. J Psychosom Res. 1996;41(4):365-76.

11. Graham CD, Rose MR, Hankins M, Chalder T, Weinman J. Separating emotions from consequences in muscle disease: Comparing beneficial and unhelpful illness schemata to inform intervention development. J Psychosom Res. 2013;74(4):320-6.

12. Graham CD, Gouick J, Krahé C, Gillanders D. A systematic review of the use of Acceptance and Commitment Therapy (ACT) in chronic disease and long-term conditions. Clin Psychol Rev. 2016;46:46-58.

13. Engel GL. The need for a new medical model: a challenge for biomedicine. Holistic Med. 1989;4(1):37-53.

14. Voet N, Bleijenberg G, Hendriks J, de Groot I, Padberg G, van Engelen B, et al. Both aerobic exercise and cognitive-behavioral therapy reduce chronic fatigue in FSHD: an RCT. Neurology. 2014 Nov 18;83(21):1914-22.

15. Graham CD, Simmons Z, Stuart SR, Rose MR. The potential of psychological interventions to improve quality of life and mood in muscle disorders. Muscle Nerve. 2015;52(1):131-6.

16. Hayes SC, Strosahl KD, Wilson KG. Acceptance and commitment therapy: An experiential approach to behavior change. Guilford Press; 1999.

For peer review only - http://bmjopen.bmj.com/site/about/guidelines.xhtml

17. McCracken LM, Morley S. The psychological flexibility model: a basis for integration and progress in psychological approaches to chronic pain management. The Journal of Pain. 2014;15(3):221-34.

18. Graham CD, Gouick J, Ferreira N, Gillanders D. The influence of psychological flexibility on life satisfaction and mood in muscle disorders. Rehabilitation psychology. 2016;61(2):210.

19. A-Tjak JG, Davis ML, Morina N, Powers MB, Smits JA, Emmelkamp PM. A meta-analysis of the efficacy of acceptance and commitment therapy for clinically relevant mental and physical health problems. Psychother Psychosom. 2015;84(1):30-6.

20. Öst L. The efficacy of Acceptance and Commitment Therapy: an updated systematic review and meta-analysis. Behav Res Ther. 2014;61:105-21.

21. Hann KE, McCracken LM. A systematic review of randomized controlled trials of Acceptance and Commitment Therapy for adults with chronic pain: Outcome domains, design quality, and efficacy. Journal of Contextual Behavioral Science. 2014;3(4):217-27.

22. Feros DL, Lane L, Ciarrochi J, Blackledge JT. Acceptance and Commitment Therapy (ACT) for improving the lives of cancer patients: a preliminary study. Psycho-Oncology. 2013;22(2):459-64.

23. Gregg JA, Callaghan GM, Hayes SC, Glenn-Lawson JL. Improving diabetes self-management through acceptance, mindfulness, and values: a randomized controlled trial. J Consult Clin Psychol. 2007;75(2):336.

24. Lundgren T, Dahl J, Melin L, Kies B. Evaluation of acceptance and commitment therapy for drug refractory epilepsy: a randomized controlled trial in South Africa—a pilot study. Epilepsia. 2006;47(12):2173-9.

25. Charova E, Dorstyn D, Tully P, Mittag O. Web-based interventions for comorbid depression and chronic illness: a systematic review. J Telemed Telecare. 2015 Jun;21(4):189-201.

26. Beatty L, Lambert S. A systematic review of internet-based self-help therapeutic interventions to improve distress and disease-control among adults with chronic health conditions. Clin Psychol Rev. 2013;33(4):609-22.

27. Hudson JL, Moss-Morris R, Game D, Carroll A, McCrone P, Hotopf M, et al. Improving distress in dialysis (iDiD): a feasibility two-arm parallel randomised controlled trial of an online cognitive behavioural therapy intervention with and without therapist-led telephone support for psychological distress in patients undergoing haemodialysis. BMJ Open. 2016 Apr 12;6(4):e011286,2016-011286.

28. Graham CD, Chalder T, Rose MR, Gavriloff D, McCracken LM, Weinman J. A pilot case series of a brief acceptance and commitment therapy (ACT)-based guided self-help intervention for improving quality of life and mood in muscle disorders. the Cognitive Behaviour Therapist. 2017;10.

29. Rose M. 188th ENMC International Workshop: Inclusion Body Myositis, 2–4 December 2011, Naarden, The Netherlands. Neuromuscul Disord. 2013;23(12):1044-55.

30. Wong A, Nyenhuis D, Black SE, Law LS, Lo ES, Kwan PW, et al. Montreal Cognitive Assessment 5-minute protocol is a brief, valid, reliable, and feasible cognitive screen for telephone administration. Stroke. 2015 Apr;46(4):1059-64.

31. Harris R. ACT made simple: An easy-to-read primer on acceptance and commitment therapy. New Harbinger Publications; 2009.

For peer review only - http://bmjopen.bmj.com/site/about/guidelines.xhtml

32. Plumb JC, Vilardaga R. Assessing treatment integrity in acceptance and commitment therapy: Strategies and suggestions. International Journal of Behavioral Consultation and Therapy. 2010;6(3):263.

33. Mayhew A, Cano S, Scott E, Eagle M, Bushby K, Muntoni F. Moving towards meaningful measurement: Rasch analysis of the North Star Ambulatory Assessment in Duchenne muscular dystrophy. Developmental Medicine & Child Neurology. 2011;53(6):535-42.

34. Cuthbert SC, Goodheart GJ. On the reliability and validity of manual muscle testing: a literature review. Chiropractic & osteopathy. 2007;15(1):4.

35. Vincent KA, Carr AJ, Walburn J, Scott DL, Rose MR. Construction and validation of a quality of life questionnaire for neuromuscular disease (INQoL). Neurology. 2007 Mar 27;68(13):1051-7.

36. Mundt JC, Marks IM, Shear MK, Greist JH. The Work and Social Adjustment Scale: a simple measure of impairment in functioning. Br J Psychiatry. 2002 May;180:461-4.

37. Zigmond AS, Snaith RP. The hospital anxiety and depression scale. Acta Psychiatr Scand. 1983;67(6):361-70.

38. Bjelland I, Dahl AA, Haug TT, Neckelmann D. The validity of the Hospital Anxiety and Depression Scale: an updated literature review. J Psychosom Res. 2002;52(2):69-77.

39. Fries JF, Spitz PW, Young DY. The dimensions of health outcomes: the health assessment questionnaire, disability and pain scales. J Rheumatol. 1982 Sep-Oct;9(5):789-93.

40. Jackson C, Barohn R, Gronseth G, Pandya S, Herbelin L. Inclusion body myositis functional rating scale: a reliable and valid measure of disease severity. Muscle Nerve. 2008;37(4):473-6.

41. Ferguson L, Scheman J. Patient global impression of change scores within the context of a chronic pain rehabilitation program. The Journal of Pain. 2009;10(4):S73.

42. Hayes SC, Strosahl K, Wilson KG, Bissett RT. Measuring experiential avoidance: A preliminary test of a working model. The psychological record. 2004;54(4):553.

43. Brown KW, Ryan RM. Perils and promise in defining and measuring mindfulness: Observations from experience. Clinical Psychology: Science and Practice. 2004;11(3):242-8.

44. McCracken L, Chilcot J, Norton S. Further development in the assessment of psychological flexibility: A shortened Committed Action Questionnaire (CAQ-8). European Journal of Pain. 2015;19(5):677-85.

45. Braun V, Clarke V. Using thematic analysis in psychology. Qualitative research in psychology. 2006;3(2):77-101.

46. Norman GR, Sloan JA, Wyrwich KW. Is it simple or simplistic? Med Care. 2003 May;41(5):599-600.

47. White PD, Sharpe MC, Chalder T, DeCesare JC, Walwyn R. Protocol for the PACE trial: A randomised controlled trial of adaptive pacing, cognitive behaviour therapy, and graded exercise as supplements to standardised specialist medical care versus standardised specialist medical care alone for patients with the chronic fatigue syndrome/myalgic encephalomyelitis or encephalopathy. BMC neurology. 2007;7(1):1.

48. World Medical Association. Declaration of Helsinki. Ethical principles for medical research involving human subjects. http://www.wma.net/e/policy/b3.htm. 2008.

For peer review only

For peer review only - http://bmjopen.bmj.com/site/about/guidelines.xhtml

49. Dixon JR. The international conference on harmonization good clinical practice guideline. Quality Assurance: Good Practice, Regulation, and Law. 1999;6(2):65-74.

FIGURE LEGEND

Figure 1: Consort diagram for ACTMuS

For peer review only

For peer review only - http://bmjopen.bmj.com/site/about/guidelines.xhtml

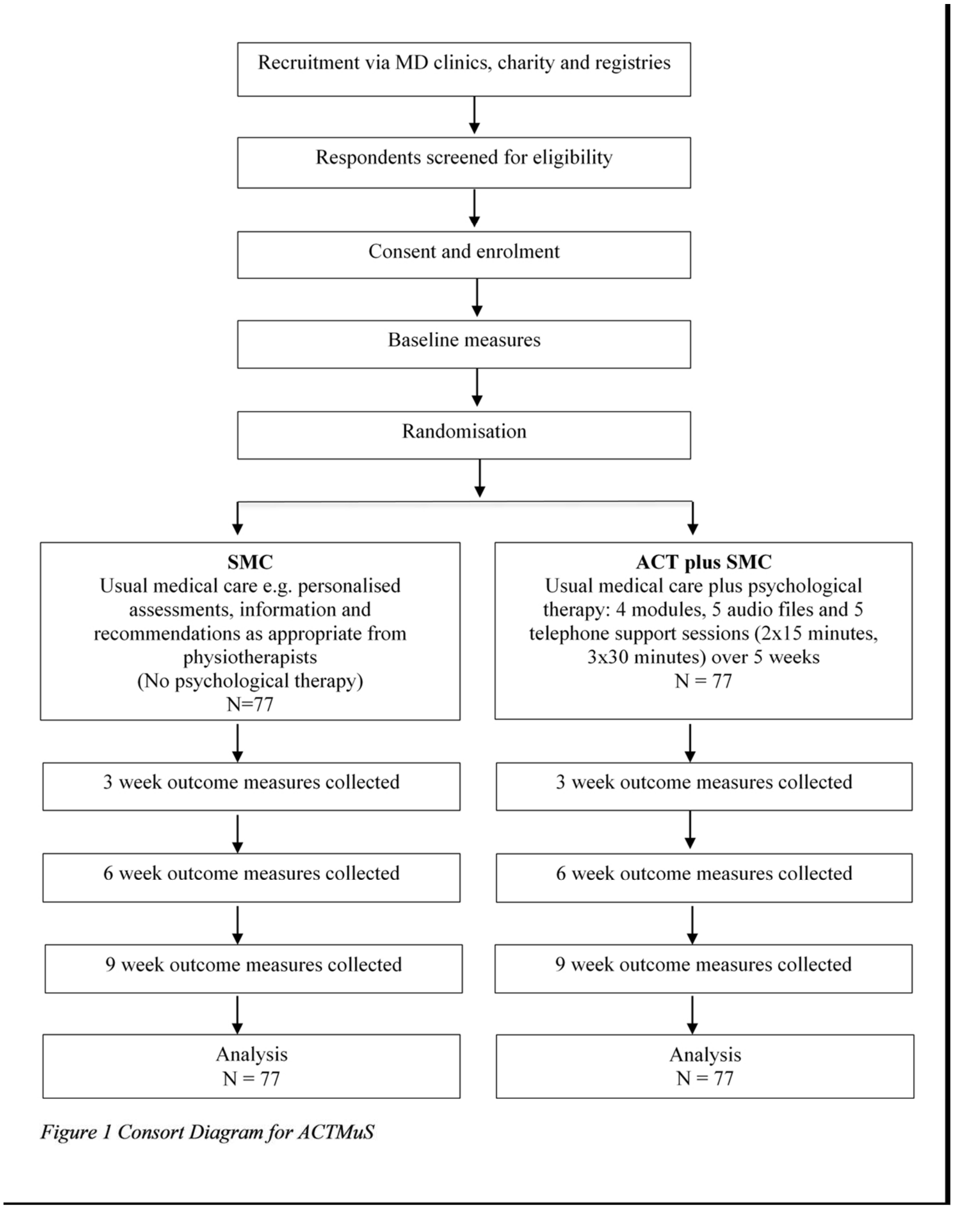

Figure 1: Consort Diagram for ACTMuS

111x141mm (300 x 300 DPI)

SPIRIT
STANDARD PROTOCOL ITEMS: RECOMMENDATIONS FOR INTERVENTIONAL TRIALS

SPIRIT 2013 Checklist: Recommended items to address in a clinical trial protocol and related documents*

Section/item	Item No	Description	Addressed on page number
**Administrative information**			
Title	1	Descriptive title identifying the study design, population, interventions, and, if applicable, trial acronym	X (p.1)______
Trial registration	2a	Trial identifier and registry name. If not yet registered, name of intended registry	X(p.15)_________ _____
	2b	All items from the World Health Organization Trial Registration Data Set	____N/A_____
Protocol version	3	Date and version identifier	X (p. 2 Title Page_Authors_Administrative Information)_____
Funding	4	Sources and types of financial, material, and other support	X (p. 2 Title Page_Authors_Administrative Information)_____ ___________
Roles and responsibilities	5a	Names, affiliations, and roles of protocol contributors	X (p. 2 Title Page_Authors_Administrative Information)_____ _________

For peer review only

	5b	Name and contact information for the trial sponsor	_X ___________
	5c	Role of study sponsor and funders, if any, in study design; collection, management, analysis, and interpretation of data; writing of the report; and the decision to submit the report for publication, including whether they will have ultimate authority over any of these activities	_____________
	5d	Composition, roles, and responsibilities of the coordinating centre, steering committee, endpoint adjudication committee, data management team, and other individuals or groups overseeing the trial, if applicable (see Item 21a for data monitoring committee)	____________
**Introduction**			
Background and rationale	6a	Description of research question and justification for undertaking the trial, including summary of relevant studies (published and unpublished) examining benefits and harms for each intervention	_X (p. 4-5)____
	6b	Explanation for choice of comparators	_X (p. 4-5)___
Objectives	7	Specific objectives or hypotheses	_X (p.5)_____
Trial design	8	Description of trial design including type of trial (eg, parallel group, crossover, factorial, single group), allocation ratio, and framework (eg, superiority, equivalence, noninferiority, exploratory)	__X (p.5)__
**Methods: Participants, interventions, and outcomes**			
Study setting	9	Description of study settings (eg, community clinic, academic hospital) and list of countries where data will be collected. Reference to where list of study sites can be obtained	___X (p.6)__
Eligibility criteria	10	Inclusion and exclusion criteria for participants. If applicable, eligibility criteria for study centres and individuals who will perform the interventions (eg, surgeons, psychotherapists)	__X (p.6)__
Interventions	11a	Interventions for each group with sufficient detail to allow replication, including how and when they will be administered	__X (p.6-8)__

For peer review only - http://bmjopen.bmj.com/site/about/guidelines.xhtml

	11b	Criteria for discontinuing or modifying allocated interventions for a given trial participant (eg, drug dose change in response to harms, participant request, or improving/worsening disease)	___________
	11c	Strategies to improve adherence to intervention protocols, and any procedures for monitoring adherence (eg, drug tablet return, laboratory tests)	______X (p.7)__
	11d	Relevant concomitant care and interventions that are permitted or prohibited during the trial	____X (p.6)__
Outcomes	12	Primary, secondary, and other outcomes, including the specific measurement variable (eg, systolic blood pressure), analysis metric (eg, change from baseline, final value, time to event), method of aggregation (eg, median, proportion), and time point for each outcome. Explanation of the clinical relevance of chosen efficacy and harm outcomes is strongly recommended	___X (p.8-10)_
Participant timeline	13	Time schedule of enrolment, interventions (including any run-ins and washouts), assessments, and visits for participants. A schematic diagram is highly recommended (see Figure)	___X (p. 11-12)_
Sample size	14	Estimated number of participants needed to achieve study objectives and how it was determined, including clinical and statistical assumptions supporting any sample size calculations	__X (p.13)__
Recruitment	15	Strategies for achieving adequate participant enrolment to reach target sample size	__X (p.13)______

Methods: Assignment of interventions (for controlled trials)

Allocation:

Sequence generation	16a	Method of generating the allocation sequence (eg, computer-generated random numbers), and list of any factors for stratification. To reduce predictability of a random sequence, details of any planned restriction (eg, blocking) should be provided in a separate document that is unavailable to those who enrol participants or assign interventions	__X (p.13)_____
Allocation concealment mechanism	16b	Mechanism of implementing the allocation sequence (eg, central telephone; sequentially numbered, opaque, sealed envelopes), describing any steps to conceal the sequence until interventions are assigned	___X (p.13)___
Implementation	16c	Who will generate the allocation sequence, who will enrol participants, and who will assign participants to interventions	____X (p.13)____

For peer review only - http://bmjopen.bmj.com/site/about/guidelines.xhtml

Blinding (masking)	17a	Who will be blinded after assignment to interventions (eg, trial participants, care providers, outcome assessors, data analysts), and how	____X (p.13)____
	17b	If blinded, circumstances under which unblinding is permissible, and procedure for revealing a participant's allocated intervention during the trial	_____X (p.13)___
**Methods: Data collection, management, and analysis**			
Data collection methods	18a	Plans for assessment and collection of outcome, baseline, and other trial data, including any related processes to promote data quality (eg, duplicate measurements, training of assessors) and a description of study instruments (eg, questionnaires, laboratory tests) along with their reliability and validity, if known. Reference to where data collection forms can be found, if not in the protocol	___X (p.13)___
	18b	Plans to promote participant retention and complete follow-up, including list of any outcome data to be collected for participants who discontinue or deviate from intervention protocols	____X (p.13)___
Data management	19	Plans for data entry, coding, security, and storage, including any related processes to promote data quality (eg, double data entry; range checks for data values). Reference to where details of data management procedures can be found, if not in the protocol	____X (p.14)___
Statistical methods	20a	Statistical methods for analysing primary and secondary outcomes. Reference to where other details of the statistical analysis plan can be found, if not in the protocol	___X (p.14)___
	20b	Methods for any additional analyses (eg, subgroup and adjusted analyses)	_____ ___
	20c	Definition of analysis population relating to protocol non-adherence (eg, as randomised analysis), and any statistical methods to handle missing data (eg, multiple imputation)	___X (p.14)____
**Methods: Monitoring**			
Data monitoring	21a	Composition of data monitoring committee (DMC); summary of its role and reporting structure; statement of whether it is independent from the sponsor and competing interests; and reference to where further details about its charter can be found, if not in the protocol. Alternatively, an explanation of why a DMC is not needed	_____________
	21b	Description of any interim analyses and stopping guidelines, including who will have access to these interim results and make the final decision to terminate the trial	_____________

Harms	22	Plans for collecting, assessing, reporting, and managing solicited and spontaneously reported adverse events and other unintended effects of trial interventions or trial conduct	__X (p. 14-15)__
Auditing	23	Frequency and procedures for auditing trial conduct, if any, and whether the process will be independent from investigators and the sponsor	_____________
**Ethics and dissemination**			
Research ethics approval	24	Plans for seeking research ethics committee/institutional review board (REC/IRB) approval	___X (p. 15)__
Protocol amendments	25	Plans for communicating important protocol modifications (eg, changes to eligibility criteria, outcomes, analyses) to relevant parties (eg, investigators, REC/IRBs, trial participants, trial registries, journals, regulators)	_____________
Consent or assent	26a	Who will obtain informed consent or assent from potential trial participants or authorised surrogates, and how (see Item 32)	___X (p.15)__
	26b	Additional consent provisions for collection and use of participant data and biological specimens in ancillary studies, if applicable	_____________
Confidentiality	27	How personal information about potential and enrolled participants will be collected, shared, and maintained in order to protect confidentiality before, during, and after the trial	______X (p.14)__
Declaration of interests	28	Financial and other competing interests for principal investigators for the overall trial and each study site	__X (p.15)____
Access to data	29	Statement of who will have access to the final trial dataset, and disclosure of contractual agreements that limit such access for investigators	____________
Ancillary and post-trial care	30	Provisions, if any, for ancillary and post-trial care, and for compensation to those who suffer harm from trial participation	____________
Dissemination policy	31a	Plans for investigators and sponsor to communicate trial results to participants, healthcare professionals, the public, and other relevant groups (eg, via publication, reporting in results databases, or other data sharing arrangements), including any publication restrictions	____X (p.15)_
	31b	Authorship eligibility guidelines and any intended use of professional writers	____________

For peer review only

	31c	Plans, if any, for granting public access to the full protocol, participant-level dataset, and statistical code	____________

Appendices

Informed consent materials	32	Model consent form and other related documentation given to participants and authorised surrogates	______________
Biological specimens	33	Plans for collection, laboratory evaluation, and storage of biological specimens for genetic or molecular analysis in the current trial and for future use in ancillary studies, if applicable	_____________

*It is strongly recommended that this checklist be read in conjunction with the SPIRIT 2013 Explanation & Elaboration for important clarification on the items. Amendments to the protocol should be tracked and dated. The SPIRIT checklist is copyrighted by the SPIRIT Group under the Creative Commons "Attribution-NonCommercial-NoDerivs 3.0 Unported" license.

For peer review only

Acceptance and Commitment Therapy for MuScle disease (ACTMuS): protocol for a two-arm randomised controlled trial of a brief guided self-help ACT programme for improving quality of life in people with muscle diseases

Journal:	*BMJ Open*
Manuscript ID	bmjopen-2018-022083.R1
Article Type:	Protocol
Date Submitted by the Author:	04-Jun-2018
Complete List of Authors:	Rose, Michael ; King's College Hospital, Department of Neurology<br>Norton, Sam; King's College London, Institute of Psychiatry, Psychology and Neuroscience, Department of Psychology<br>Vari, Chiara; King's College London, Institute of Psychiatry, Psychology and Neuroscience, Department of Psychological Medicine<br>Edwards, Victoria; King's College London, Institute of Psychiatry, Psychology and Neuroscience, Department of Psychological Medicine<br>McCracken, Lance; King's College London, Institute of Psychiatry, Psychology and Neuroscience, Department of Psychology<br>Graham, Christopher; University of Leeds, Leeds Institute of Health Sciences<br>Radunovic, Aleksandar; Royal London Hospital, Barts and the London MND Centre<br>Chalder, Trudie; King's College London, Institute of Psychiatry, Psychology and Neuroscience, Department of Psychological Medicine
**Primary Subject Heading**:	Neurology
Secondary Subject Heading:	Neurology
Keywords:	Muscle diseases, Quality of life, Acceptance and commitment therapy, Randomised control trial, Adult

Title: Acceptance and Commitment Therapy for MuScle Disease (ACTMuS): Protocol for a two-arm randomised controlled trial of a brief guided self-help ACT programme for improving quality of life in people with Muscle diseases

Authors: Rose MR, Norton S, Vari C, Edwards V, McCracken L, Graham C.D., Radunovic A, Chalder T. On behalf of the ACTMUS team

Michael R. Rose (corresponding author)

King's College Hospital,
Department of Neurology,
Denmark Hill,
London,
SE5 9RS.

m.r.rose@kcl.ac.uk

Telephone: 020 3299 8343

Michael R. Rose, King's College Hospital, Department of Neurology, London, UK

Sam Norton, King's College London, Institute of Psychiatry, Psychology and Neuroscience, Department of Psychology, London, UK

Chiara Vari, King's College London, Institute of Psychiatry, Psychology and Neuroscience, Department of Psychological Medicine, London, UK

Victoria Edwards, King's College London, Institute of Psychiatry, Psychology and Neuroscience, Department of Psychological Medicine, London, UK

Lance McCracken, King's College London, Institute of Psychiatry, Psychology and Neuroscience, Department of Psychology, London, UK

Christopher D. Graham, University of Leeds, Leeds Institute of Health Sciences, Leeds, UK

Aleksandar Radunovic, Royal London Hospital, Barts and the London MND Centre, London, UK,

Trudie Chalder, King's College London, Institute of Psychiatry, Psychology and Neuroscience, Department of Psychological Medicine, London, UK

Key words: Muscle diseases; quality of life; acceptance and commitment therapy; randomised control trial; adults

Trial registration number: NCT02810028 (ClinicaTrials.gov).

Trial status: Ongoing recruitment.

Protocol version: Version 11 (04/04/2017)

Funding: National Institute for Health Research (NIHR) Research for Patient Benefit grant (Reference number: PB-PG-061331085). Muscular Dystrophy UK have been partners from the commencement of the study and funded the trial therapist.

The trial is sponsored by King's College Hospital NHS Foundation Trust and co-sponsored by King's College London.

For peer review only

For peer review only - http://bmjopen.bmj.com/site/about/guidelines.xhtml

Roles and responsibilities:
MR, CG, SN, LM, AR and TC obtained funding for this study. MR, SN, CV, VE, LM, CG, AR and TC contributed to the study protocol. MR and TC are responsible for overall supervision. All authors read, contributed towards and approved of the final manuscript.

Word Count: 5296

For peer review only

For peer review only - http://bmjopen.bmj.com/site/about/guidelines.xhtml

ABSTRACT

Introduction: In adults, muscle disease (MD) is most often a chronic long term condition with no definitive cure. It causes wasting and weakness of the muscles resulting in a progressive decline in mobility, alongside other symptoms, and is typically associated with reduced quality of life (QoL). Previous research suggests that a psychological intervention, and in particular Acceptance and Commitment Therapy (ACT), may help improve QoL in MD. ACT is a newer type of cognitive behavioural treatment that aims to improve QoL by virtue of improvement in a process called psychological flexibility. The primary aim of this randomised control trial (RCT) is to evaluate the efficacy of a guided self-help ACT programme for improving QoL in people with MD. Main secondary outcomes are mood, symptom impact, work and social adjustment and function at 9-week follow-up.

Methods and analysis: ACTMuS is an assessor-blind, multicentre, two-armed, parallel-group RCT to assess the efficacy of ACT plus standard medical care (SMC) compared to SMC alone. Individuals with a diagnosis of one of four specific MDs, with a duration of at least 6 months, and with mild to moderate anxiety or depression (HADS score ≥ 8) will be recruited from UK-based MD clinics and MD patient support organisations. Participants will be randomised to either ACT plus SMC or SMC alone by an independent randomisation service. Participants will be followed up at 3, 6 and 9 weeks. Analysis will be intention to treat, conducted by the trial statistician who will be blinded to treatment allocation.

Ethics and dissemination: The study has received full ethical approval. Study results will be disseminated via peer-reviewed publications, conference presentations and journal articles. Data obtained from the trial will enable clinicians and health service providers to make informed decisions regarding the efficacy of ACT for improving QoL for patients with MD.

For peer review only

STRENGTHS AND LIMITATIONS OF THIS STUDY:

- ACTMuS is the first randomised controlled trial to test the efficacy of an ACT-informed intervention for MDs against standard medical care (SMC).
- To our knowledge this is the largest trial of a psychological intervention for MD, and the first that primarily targets QoL.
- In order to improve access and increase uptake, the intervention is brief and designed to be delivered remotely (via e-mail and telephone).
- Only a short follow-up period was included and consequently this study will not be able to investigate longer-term treatment effects. An intrinsic limitation to the study is the lack of comparable therapist's time and attention within the SMC alone group.

For peer review only

For peer review only - http://bmjopen.bmj.com/site/about/guidelines.xhtml

INTRODUCTION

Background and rationale

Muscle diseases (MDs) are a group of primary disorders of the muscle, the majority of which are chronic and progressive. They affect approximately over 70,000 people (children and adults) in the UK (1). The diagnosis of a specific MD is based on several criteria including the pattern of the muscle weakness, the muscle biopsy appearances and in some cases genetic testing. Four MDs, namely limb girdle muscular dystrophy (LGMD), Becker's muscular dystrophy (BMD) (dystrophin deficiency), facioscapulohumeral muscular dystrophy (FSHD), and inclusion body myositis (IBM) comprise a significant proportion of those seen in an adult muscle clinic. They are characterised by progressive limb muscle wasting and weakness that cause difficulties with mobility and physical functioning. Some MDs also involve bulbar muscles causing dysphagia, respiratory muscles causing respiratory compromise, and cardic muscle causing cardiac symptoms. As well as weakness, commonly reported symptoms include chronic pain, sleep disturbance and fatigue (2, 3). Unlike some MDs these four MDs do not have additional multi-system or central nervous system involvement. At present most MDs are without disease-modifying treatments or cures.

Typically people with MD report reduced quality of life (QoL) and may experience increased levels of anxiety and depression (4, 5). A systematic review of 26 studies highlighted that, compared to controls, people with MD had compromised QoL in all areas of functioning (5). The degree of physical disability is a determinant of QoL in MDs (5). Therefore, clinical treatment is often directed at maintaining physical functioning. For example, physiotherapy and occupational therapy are applied to maximise remaining muscle function and patients are monitored for any cardiac or respiratory problems that may need supportive treatment. Even so, such interventions may have ability to improve QoL to a degree.

However,physical disability does not explain all the variance in QoL (4, 6, 7). Psychological variables such as illness beliefs (6, 8), coping methods (9-11) and psychological flexibility (12) have been shown to account for variance in QoL. These findings are in line with a biopsychosocial model (13) where QoL is theorised to depend upon the interaction of psychological factors with biological and social factors.

The relationship between QoL and psychosocial factors in MD provides a treatment opportunity. In the absence of a direct cure for MD, psychological interventions may offer a way to enhance QoL. Encouragingly, a recent small RCT of a traditional cognitive behavioural intervention for improving fatigue in FSHD showed a beneficial effect on fatigue and QoL (14).

Acceptance and Commitment Therapy (ACT)

Dependent on treatment aims, several types of psychological interventions may be helpful for people with MD (15). ACT is a newer form of cognitive-behavioural approach that focuses on improving a process called psychological flexibility (16). Psychological flexibility is "[…] the capacity to persist or to change behaviour in a way that includes conscious and open contact with thoughts and feelings, appreciates what the situation affords, and serves one's goals and values" (17). In observational studies psychological flexibility has been shown to be predictive of positive outcomes (QoL, mood) and better functioning across a range of challenging contexts, such as living with chronic illness and chronic pain. A 4-month prospective observational study with a group of people with MD observed that psychological flexibility was predictive of change in life satisfaction and anxiety (18).

Given its focus on improving psychological flexibility, ACT may prove beneficial for those with MD. In ACT a number of treatment techniques are used to improve psychological flexibility including therapist interaction, mindfulness and functional analysis. These aim to: a) help individuals become more aware of thoughts and feelings and their relationship to, in this case, their MD; b) broaden the range of behaviours (often to include open and willing responses) that can be applied when in the presence of difficult thoughts and feelings; c) help them to flexibly choose those behaviours that help them to make progress on their over-arching goals and values.

Several recent meta-analyses have suggested that ACT is an efficacious treatment for improving mood and QoL in mental health conditions (19, 20) and chronic pain (21). In chronic diseases there is a growing evidence base (12), with encouraging results for trials with cancer (22), diabetes (23), and epilepsy (24)

For peer review only - http://bmjopen.bmj.com/site/about/guidelines.xhtml

populations. However, to date, most studies of ACT for chronic diseases have been of a preliminary nature (i.e. small, uncontrolled, and/or lower quality (12). Thus, adequately-powered, rigorous evaluation of ACT for improving QoL in chronic diseases will be a helpful addition to the literature.

The selection of a guided self-help approach

The one comprehensive trial of a psychological intervention in MD (14) tested the efficacy of a 16 session traditional cognitive behavioural therapy for improving fatigue. While results were encouraging, both on fatigue and secondary outcomes such as QoL and mood, for several reasons (including the time spent travelling) uptake was low, and a proportion (24%) struggled to achieve even a minimal level of adherence to the intervention. This suggests that issues, perhaps mobility difficulties and treatment length, may hinder face-to-face treatment options for many with MD. A further problem with the development and implementation of any new treatment concerns the resource limitations common to many health service providers, including the NHS in the UK.

A practical solution to this problem could be the implementation of an intervention delivered remotely through online and computerised self-guided resources aided by telephone or video calls. Such methods of delivery are increasingly feasible given that the majority of UK households now have web access. Also recent systematic reviews suggest that promising results can be obtained from web-based interventions for adults with chronic illnesses and depression (25-27). Web based interventions not only remove the burden of travel, but also allows the completion of material at a convenient place, time, and pace. Remotely delivered interventions can be a cost-effective approach for providing treatment increasing its chances of wider implementation.

Given the potential benefits of using ACT within an MD population, and taking into account potential barriers to participation, we developed,for this population,a brief self-guided intervention that can be supported remotely via e-mail and telephone contact with a therapist. We assessed this in a preliminary multiple-baseline case series with seven participants (28). The results of this case series suggested that such an approach was practicable and acceptable and that mood and QoL might respond to this treatment. Given this promising evaluation we plan to conduct a randomised controlled trial, to evaluate the efficacy of this intervention for improving QoL in people living with MD.

This protocol describes a multicentre, two-armed, parallel-group RCT to assess the efficacy of self-guided ACT in improving QoL in individuals with MDs. A total of 154 people referred from MD clinics, Muscular Dystrophy UK and specific MD registries will be randomly allocated to receive either self-guided ACT plus standard medical care (SMC) or SMC alone. We hypothesise that the group who receive self-guided ACT plus SMC will report an improved QoL at the primary end point of 9 weeks follow-up compared with those receiving SMC alone.

Objectives

Primary objective:

1) The primary objective is to evaluate the efficacy of ACT plus SMC on QoL compared to SMC alone at 9-weeks.

Secondary objectives:

1) To evaluate the impact of ACT plus SMC compared to SMC alone on secondary outcomes: mood, symptom impact domains, work and social adjustment and function at 9-week follow-up.
2) To assess the processes that may be responsible for mediating the treatment effect.
3) To evaluate participants' views of the intervention and factors that act as facilitators and barriers to participation via a nested qualitative study.

Trial Design

ACTMuS is an assessor-blind, multicentre, two-armed, parallel-group randomised controlled trial (RCT)

For peer review only - http://bmjopen.bmj.com/site/about/guidelines.xhtml

METHODS: PARTICIPANTS, INTERVENTIONS AND OUTCOMES

Setting

A total of 154 participants will be recruited from UK National Health Service MD clinics, Muscular Dystrophy UK (MD-UK) and 3 disease-specific registries (FSHD, dysferlin, IBM). After obtaining consent, the baseline assessments will take place in MD clinics at King's College Hospital (NHS Foundation Hospital Trust), The Royal London Hospital (Barts Health NHS Trust) and University Hospital Southampton (NHS Foundation Trust). The remainder of the study including treatment will be conducted remotely via email, telephone or video calls.

Eligibility criteria

Inclusion criteria

1) Adults (aged 18 years and over) diagnosed with one of the following MDs that has been present for greater than 6 months:
    a. One of the limb girdle muscular dystrophies (LGMD) genetically or biopsy proven,
    b. Becker' muscular dystrophy (BMD) (dystrophin deficiency) with biopsy or genetic diagnosis,
    c. Facioscapulohumeral muscular dystrophy (FSHD) diagnosed clinically with specific genetic abnormality in the subject or their family,
    d. Inclusion body myositis (IBM) clinic-pathologically defined, clinically defined or probable IBM based on European Neuromuscular Centre ENMC research diagnostic criteria 2013 (29).
2) Potential participants must have access to the Internet and a computer to receive the study materials
3) HADS score of ≥ 8 for depression or ≥ 8 for anxiety.

Exclusion criteria

1) Unstable complications of MDs including neuromuscular respiratory weakness or cardiomyopathy.
2) Major active co-morbidities unrelated to MD (such as arthritis, respiratory disease, cardiovascular disease).
3) Current diagnosis of an active major mental health disorder likely to interfere with participation
4) Current or recent participation in other treatment intervention studies (< 4 weeks after completion).
5) Currently receiving psychological support or psychotherapy.
6) Inability to read English questionnaires
7) Cognitive impairment that would prevent comprehension of ACT modules and questionnaires (as assessed by the Montreal Cognitive Assessment 5-minute protocol, (30)).

INTERVENTIONS

Acceptance and Commitment Therapy

The intervention is a remotely supported, guided self-help ACT programme (developed mainly by CG and LM, informed by other sources (16, 22, 31). It consists of four modules and corresponding audio files supported by five telephone support sessions with the trial therapist (Table 1). The first three modules are expected to take the participant 1 hour 30 minutes each and the final module 45 minutes to complete. This includes time to read the modules, listen to the audio files and work through tasks and exercises. During the first call, prior to receiving the first module (either by telephone or video call), the therapist will provide a 15-minute introduction then email the first module. The subsequent three phone calls will each last 30-minutes and will be followed by the next module. The telephone sessions provide participants with a chance to discuss the modules they have read, including their experiences of the exercises, and receive help with difficulties encountered. A 15-minute phone call will follow the final module to conclude the treatment.

For peer review only - http://bmjopen.bmj.com/site/about/guidelines.xhtml

Module 1: Mindfulness and unhooking	• Normalising difficult thoughts and feelings, given the context of MD, and associated struggles.<br>• Discussion of consequences from trying to get rid of unwanted thoughts and feelings and opportunities for alternative responses.<br>• Introducing other ways to interact with unwanted thoughts and feelings:<br>1. Mindfulness or learning to be present in the moment and experience thoughts and feelings in a non-judgemental way.<br>2. Unhooking from thoughts or learning ways to step-back from thoughts that often restrict actions or lead to avoidance.<br>• Homework 1: Daily diary of mindfulness practice.<br>• Homework 2: Identifying where one struggles and experimenting with other responses.
Module 2: Follow your values	• Identifying values and ways to pursue them with MD. Exploring the link between difficult thoughts and feelings and one's goals and values.<br>• Homework 1: Continuation of daily mindfulness practice<br>• Homework 2: Setting goals in the context of values, experimenting with taking the 'smallest possible step' which is consistent with ones values, noticing the thoughts and feelings that occur in the context of values activity.
Module 3: Take an observer perspective	• Experimenting with taking an observer perspective on one's experiences.<br>• Considering labels one attaches to oneself (especially given the context of MD). Noticing a choice over buying into labels, and the impact of labels over one's behaviour.<br>• Introducing a more flexible approach to mindfulness<br>• Homework 1: Continuation of daily mindfulness practice with addition of flexible attention and noticing the observer self<br>• Homework 2: Continuation of valued activities
Module 4: Recap, reflection and suggestions for staying committed	• Review of homework tasks and skills learnt.<br>• Self-identification of effective and ineffective behaviour patterns.<br>• Goal planning and normalisation of set-backs with a compassionate approach to getting back on track.

Table 1 Summary of ACT modules

The Clinical Psychologist delivering the ACT support sessions will attend a three-day training session prior to the start of recruitment. The first day will cover the rationale behind the study and insights into MD diagnosis, symptoms and impact on QoL (led by MR, a Consultant Neurologist). Details of the study protocol will be reviewed as well as the role of the therapist, standard operating procedures, recording and storage of the support sessions and confidential information, deviations, dropouts and adverse event procedures (led by TC, Professor of Cognitive Behaviour Therapy). The ACT training including presentations, exercises and role-plays will last 2 days. This will be led by LM, a Clinical Psychologist and expert in ACT, assisted by CG, an ACT-trained Clinical Psychologist. Obstacles to patient engagement with ACT as well as the difficulties of delivering the sessions remotely and within a brief time frame will be discussed. The therapist will attend monthly supervision meetings with a clinical supervisor (LM) to develop their skills and to ensure therapy fidelity and adherence to trial protocol. The therapist's treatment competence will be periodically assessed with an ACT-fidelity scale. This will inform monthly supervision sessions between therapist and clinical supervisor throughout the trial.

Treatment fidelity

All ACT support sessions will be audio-recorded to assess treatment fidelity. At the end of the trial a random sample of recordings will be analysed for overall fidelity by two independent researchers. A fidelity measure is currently being developed by CG and LM. This comprises a basic therapeutic competence scale and an ACT-specific fidelity scale (ACT-FS) informed by guidelines provided by Plumb and Vilardaga (32).

For peer review only - http://bmjopen.bmj.com/site/about/guidelines.xhtml

Standard Medical Care (SMC)

All participants will receive SMC, which involves the continuation of any current medical practices (without any psychological therapy). Standard medical care would be 1) review of functional impairment arising from their muscle weakness and measures suggested to ameliorate the resulting disability with home adaptations and assistive devices, 2) monitoring for respiratory or cardiac complications and appropriate referral for their management if required, 3) recommendations for local physiotherapy, 4) recommendations for falls management where appropriate, 5) answering queries about their condition usually using information leaflets from the national organisation Muscular Dystrophy UK or from disease support groups.

Baseline measures

Participants will complete baseline questionnaires (see Table 2) at the MD clinics with the research assistant (RA) and objective assessments with a physiotherapist. Participants will also complete a participant details form to provide demographic data as follows: age, gender, ethnicity, occupation status, education level, marital status, living arrangements and dependants. Clinical information will also be collected, including age of onset and duration of MD, additional diagnoses, experience of psychological therapies as well as prior treatment of anxiety or depression. Adverse events will be recorded at each assessment point of the trial.

The objective assessments conducted by physiotherapists at baseline are as follows:

- Adult Ambulatory Neuromuscular Assessment (ANA) is an adult version of the North Star Ambulatory Assessment (33) that measures motor function. Items are scored from 0-2, with an overall possible range of scores from 0-34. The higher the score, the better the patient can function. An additional item of the ANA that shall be used is the 6-minute timed walk test (6MTWT) which measures mobility by counting the distance walked within a 6-minute time-frame along with pauses or stops before the time is complete. The higher the score the greater the function of the patient.

- Manual Muscle Strength Testing (MMST; aka Manual Muscle Testing, MMT) measures the strength of 12 different muscles on both right and left from 0-5 (no contraction palpable – normal strength). There is a total possible range of scores from 0- 120, with higher scores representing levels of strength closest to normal levels (34).

Outcome measures

The RA blind to participant allocation will be responsible for ensuring outcome measures are completed at 3, 6 and 9 weeks by all participants. The link to the measures will be sent to participants via email. If participants do not complete the questionnaires by the due date they will be reminded by the RA. All time points will be taken into account during analysis but the primary efficacy end-point is the 9-week follow-up.

Primary outcome:

- The Individualised Neuromuscular Quality of Life Questionnaire (INQoL) – life area domains (section 2) is the primary outcome. The INQoL is a 10-domain, validated MD specific questionnaire to measure QoL (35). We will exclude the one domain that addresses the symptom of mytonia as none of the MDs being recruited has this symptom. This will leave 9 domains for the purpose of this study. The primary outcome of this study constitutes section 2 of the INQOL which assesses 5 life area domains (activities, independence, social functioning, emotional functioning & body image). Participants respond using a 7-point Likert scale, with higher scores indicating greater symptom impact. The primary outcome measure for this study will therefore be the change in the overall QoL score, derived from these five life domain scores, from baseline up to 9-weeks.

For peer review only - http://bmjopen.bmj.com/site/about/guidelines.xhtml

Secondary outcome measures:

- Individualised Neuromuscular Quality of Life Questionnaire (INQoL) symptom impact domains. This refers to categories within Section 1 of the INQoL (weakness, pain, tiredness) and follows the same format as described above.

- Work and Social Adjustment Scale (WSAS) is a measure of impairment at work, home and social functioning (36). The total score comprises the sum of responses to five questions, each of which is on a nine-point Likert scale. The possible range of scores is 0-40; with higher scores indicating more greatly impaired daily functioning.

- Hospital Anxiety and Depression Scale (HADS) is a self-report questionnaire to measure mood (37). It consists of fourteen items, relating to depression (7 items) and anxiety (7 items). Higher scores indicate either greater anxiety or depression. Each of the items are rated from 0-3 thus the possible range of scores for each is 0-21, with a cut-off of 8 showing good sensitivity and specificity for possible cases (38).

- Stanford Health Assessment Questionnaire Disability Index (HAQ-DI) is a measure of functional impairment and level of disability (39).There are different ways to score the HAQ-DI. As with previous studies with MD populations (18) the alternative scoring method shall be used which focuses on measuring disability not adaption by not incorporating use of aids and devices. The measure includes eight activity domains (dressing, arising, eating, walking, hygiene, reach, grip, and activities). There are 20 questions in total, each scored from 0-3. All the domains are summed for a total score with a higher score representing a greater impairment in ability to function in daily life. As additional measure of functional impairment, not treated as an efficacy outcome in the analysis, the IBM Functional Rating Scale (40) will be completed only by those participants with a diagnosis of Inclusion Body Myositis. This is a 10-point functional rating scale, with higher scores depicting greater functional impairment.

- Patient Global Impression of Change scale (PGIC) is a single item measure of a patient's impression of change over the study (41). Participants rate their impression of change on a scale of 1–7 as follows: 'very much better', 'much better', 'a little better', 'about the same', 'a little worse', 'much worse' and 'very much worse'.

- Rating of satisfaction is a single item measure of a patient's satisfaction with the outcome of the trial. Participants rate their satisfaction on a scale of 1–7 as follows: 'very satisfied', 'moderately satisfied', 'slightly satisfied', 'neither', 'slightly dissatisfied', 'moderately dissatisfied' and 'very dissatisfied'.

Putative Mediators and process variables:

- Acceptance and Action Questionnaire (AAQ-II) (42) measures psychological flexibility, including experiential avoidance and psychological inflexibility. Each of the seven items are rated on a scale of 1-7, with higher scores representing greater experiential avoidance and immobility.

- Mindfulness Attention Awareness Scale (MAAS) measures dispositional open awareness of and attention to the present moment (43). There are 15 items, each scored from 1-6. A mean of the 15 items is computed; with higher scores reflect higher levels of dispositional mindfulness.

- Committed Action Scale (CAQ) measures committed action i.e. goal-directed, flexible persistence (44). Respondents rate the extent to which the 8 items apply to them from 0-6 with a greater total score representing a greater general propensity to persist in goal-directed behaviour

For peer review only - http://bmjopen.bmj.com/site/about/guidelines.xhtml

Qualitative component

KEY

A nested qualitative study will explore patients' experience of the ACT intervention. This will help identify factors that facilitate or impede adherence and acceptability of the treatment, and perhaps give insight into treatment mechanisms. Semi-structured interviews will be conducted post-intervention with approximately 15 participants (purposively sampled to encompass a mix of gender, ages and symptom severity). Interviewers will use topic guides with open-ended questions and prompts to elicit participants' accounts of their experiences. The interviews will be transcribed verbatim. Analysis will commence after the completion of the first interview in an iterative process to allow exploration of early insights in later interviews. Standard thematic analysis techniques will be applied (45), for example, coding the data and identifying themes that capture key concepts and processes. The results will supplement quantitative results and inform any future implementation of the intervention.

Participant timeline

Information is provided in the consort diagram (Figure 1) and the screening and data schedule of assessments (Table 2). The RA will contact potentially interested participants to ensure they are fully informed and eligible to take part in the trial. If this is the case, they will be asked to sign a consent form, enrolled onto the study, and provided with a unique identification number. The baseline assessments will be in person at one of the MD clinics with the RA and physiotherapist. Following baseline, the participant will be randomised and informed by the trial coordinator as to whether they will receive ACT plus SMC or SMC alone. The participant will complete follow-up measures at 3, 6 and 9 weeks online via an email link.

Table 2 Screening and data collection across the trial: summary of key trial processes

Process	*Screening*	*Baseline*	*3 week*	*6 week*	*9 week*	*Ongoing or during trial*	*Ref*
Eligibility	X						-
Medical confirmation	X						-
HADs	X	X	X	X	X		37
MoCA	X						28
Participant details form		X					-
INQoL		X	X	X	X		33
WSAS		X	X	X	X		34
HAQ-DI		X	X	X	X		37
*IBM**		X	X	X	X		38
AAQ-II		X	X	X	X		40
MASS		X	X	X	X		41
CAQ		X	X	X	X		42
Health Events		X	X	X	X		-
ANA		X					31
6MTWT		X					31
MMST		X					32
PGIC			X	X	X		39
Satisfaction rating			X	X	X		-
Safety events (e.g. AE, SAEs)						X	-
Drop-out / withdrawal						X	-
Therapist ratings						X	-

For peer review only

**HADS**	Hospital Anxiety and Depression Scale
**MOCA**	Montreal Cognitive Assessment 5-minute protocol
**INQOL**	Individualised Neuromuscular Quality of Life Questionnaire
**WSAS**	Work and Social Adjustment Scale
**HAQ-DI**	Stanford Health Assessment Questionnaire Disability Index
**IBM**	IBM Functional Rating Scale
**AAQ-II**	Acceptance and Action Questionnaire
**MASS**	Mindfulness Attention Awareness Scale
**CAQ**	Committed Action Questionnaire
**ANA**	Adult Ambulatory Neuromuscular Assessment
**6MTWT**	6-minute timed walk test
**MMST**	Manual Muscle Strength Testing
**PGIC**	Patient Global Impression of Change scale

Sample size

154 patients will be recruited in total, 77 per trial arm. This sample size will provide adequate power to detect a standardised mean difference of 0.5 (medium magnitude) in the primary outcome between the treatment and control groups at the end of intervention assessment (5% significance, 80% power), assuming attrition of 20%. Using data from Vincent et al., (35) this equates to between an 11.5 and 15.8 point difference on each of the INQOL domains. While no information is currently available regarding clinically important differences for the INQOL, an effect size of this magnitude typically relates to a clinically important difference for health related quality of life instruments (46).

Recruitment

Participants will be recruited from MD clinics, Muscular Dystrophy UK and MD registries. In MD clinics, consultants and physiotherapists will identify potentially eligible participants who will either be given an information pack consisting of an invitation letter, information sheet and reply slip to state their interest. MD UK and MD registries will advertise the study and provide invitation packs. When someone states their interest, the RA will call them to provide further information, answer any questions and conduct screening. If they are eligible for the study (including medical confirmation from their health professional) then the participant will sign a consent form before being enrolled into the study.

Patient and Public Involvement

Dr Rose's direct work with patients within a MD clinic led to the development of the trial. In developing the study material, a small pilot study of the self-guided intervention was tested and subsequently refined in response to participants' feedback by Dr Graham (Graham, 2012). Our patient representative has been actively involved at every stage from assessing the intervention to reviewing the outcome measures. Muscular Dystrophy UK (MD UK), a patient support organisation contributed to the development of the study and will help with the referral of patients into the study. MD UK will also be involved in disseminating study results.

For peer review only - http://bmjopen.bmj.com/site/about/guidelines.xhtml

METHODS: ASSIGNMENT OF INTERVENTIONS

Allocation

Randomisation will be conducted by an independent randomisation service at the UK Clinical Research Collaboration registered at the King's Clinical Trials Unit. Randomisation will be at the level of the individual, using block randomisation with randomly varying block sizes stratified by recruiting centre. Allocation confirmation will be generated automatically and sent to unblinded research members, including the trial co-ordinator and clinical psychologist.

Allocation concealment

A position of equipoise will be maintained but blinding of the patients and therapist to the treatment condition will not be possible. The RA contacting participants and inputting data will be blind to group allocation to reduce the risk of introducing bias. To protect against unblinding, all follow-up questionnaires will be completed by participants on Bristol Online Survey (BOS) which is specifically designed for academic research and fully compliant with UK data protection law. All questionnaires will be identical for both arms and designed not to reveal treatment allocation. The trial statistician will be blind to treatment group when conducting the main data analysis.

METHODS: DATA COLLECTION, MANAGEMENT AND ANALYSIS

Data collection Methods

Baseline data will be collected prior to randomisation and consist of self-report questionnaires and objective assessments with the physiotherapist. Follow-up data (3, 6 & 9 weeks) include self-report questionnaires that will be completed remotely. At each follow-up point participants will be emailed a link to BOS along with their unique identification number. If participants are unwilling or unable to complete all the questionnaires they will be given the option of going through them on the phone or a reduced questionnaires pack with the primary outcomes (INQoL).

Data Management

All trial data will be stored in line with the Data Protection Act 1998 and in compliance with Good Clinical Practice and King's College London data management procedures. Any identifying data will be anonymised through use of unique identification numbers separated from identifying data. Data will be entered onto password-protected trial databases either on a shared hard-drive accessible only to assigned team members or in a locked cabinet in a room only accessible to staff with designated access cards. All source documents will be retained for a period of 10 years following the end of the study.

Statistical methods

Statistical analyses of the primary and secondary outcomes will be conducted after the database has been locked, with no interim analyses. All analyses will adopt the intention to treat principle (ITT). The main statistical analyses will estimate the difference in mean outcomes between patients randomised to ACT plus SMC and SMC alone at the primary endpoint of 9 weeks and at the two other post-randomisation time points. Group difference estimates, associated confidence intervals and standardised effect sizes will be reported. Significance testing will be performed where the significance level will be 5% (2-sided). Sensitivity analyses will be used to assess the robustness of conclusions to missing outcome data using a pattern mixture model approach and to departures from randomised treatment in a per protocol analysis

For peer review only - http://bmjopen.bmj.com/site/about/guidelines.xhtml

excluding those not receiving. Loss to follow-up, departures from randomised treatment and the prevalence of serious adverse events will be reported.

The primary outcome is the INQoL quality of life (QoL) score. Quality of life at all three post-randomisation time points (3, 6 and 9 weeks after randomisation) will be modelled simultaneously using a linear mixed-effects model. A random intercept will account for the non-independence of observations across individuals. Trial arm and indicator coded time will be included as covariates along with arm by time interaction terms to allow the treatment effect to vary at each time point. Also, included in the model will be the baseline level of the QoL score and a stratification indicator variable for centre. Missing data at follow up assessments is allowed where at least one observation per participant is available under the missing at random assumption.

Secondary patient outcomes (3, 6 and 9 weeks after randomisation) relating to symptom severity and functioning (WSAS; HAQ-DI), distress (HADS), impression of change (PGIC), and putative mediators of the intervention (CAQ; AAQ-II; MAAS) will be analysed using linear mixed-effects models. Random effects for the intercept and time will be included in the model.

METHODS: MONITORING

Data monitoring

The study will be monitored and audited in accordance with King's College Hospital NHS Foundation Trust and King's College London procedures. All trial related documentation will be made available on request to the sponsors, HRA/ REC and other licencing bodies. Monitoring of this study to ensure compliance with Good Clinical Practice and scientific integrity will be managed by the study team and reviewed by an independent Trial Steering Committee (TSC) & Data Monitoring & Ethics Committee (DMEC).

Harms/ Assessment of safety

Adverse events will be recorded at baseline, 3, 6 and 9 weeks. We define adverse events as 'any clinical change, disease or disorder experienced by the participant during their participation in the trial, whether or not considered related to the use of treatments being studied in the trial' (47). Adverse events will also include any other events that might have affected the health status of a participating patient (e.g. increased work stress).

The team will also monitor serious adverse events (SAEs), serious adverse reactions (SAR), suspected unexpected serious adverse reactions (SUSAR) and active withdrawals from treatment. A SAE constitutes any adverse event or reaction that: results in death or a new persistent or new significant disability or incapacity, is life threatening or requires hospitalisation, any new episode of deliberate self-harm or any other important medical condition which may jeopardise the participant and require medical or surgical intervention to prevent one of the outcomes listed above.

A serious adverse reaction (SAR) can be defined as a SAE as a reaction to the intervention. A SUSAR is any serious adverse reaction that is suspected to be caused by the intervention but was not expected. All SAEs, SARs and SUSARs will be reported immediately by the Chief Investigator to the study Sponsor and Research Ethics Committee. The TSC-DMEC will be responsible for the independent investigation of SAEs.

ETHICS AND DISSEMINATION

Research ethics approval

The trial is subject to Health Research Authority (HRA) approval that brings together the assessment of governance and legal compliance with the independent Research Ethics Committee (REC) approval. The trial has ethical approval by London - Camberwell St Giles REC (16/LO/0609). Site-specific confirmation of capacity and capability has been given by local research and development departments. The study will comply with the principles of the Declaration of Helsinki (1996) (48), the International Conference for

For peer review only - http://bmjopen.bmj.com/site/about/guidelines.xhtml

Harmonisation of Good Clinical Practice (ICH GCP; (49)) guidelines and the Research Governance Framework for Health and Social Care 2nd Edition (2005). The trial is sponsored by King's College Hospital NHS Foundation Trust (lead) and King's College London and has been registered onto a publicly accessible database (Registration number: NCT02810028).

Consent

Inclusion in the trial is appropriate only if potential participants are aged 18 or older and are able to give written informed consent. All potentially eligible participants will be fully informed about the study procedures by a participant information sheet that states participation is voluntary and that they are free to withdraw from the study at any time without giving a reason and without their care being affected.

Declaration of interests

The authors declare that they have no competing interests.

Dissemination

Summaries of findings will be offered to MD charities, registries, and support groups as well as to the wider public. The results of the trial will be communicated to participants via a newsletter. Any results from the trial will enable informed decisions regarding the provision of care for patients with MD.

CONCLUSION

Experiences of psychological distress and reduced QoL are common in people with MD. A brief, guided self-help ACT intervention with telephone support sessions has been designed for remote delivery, offering a potential solution to mobility issues inherent with MD and under-resourced healthcare services. This is the first study to evaluate whether ACT can help improve QoL for individuals with MD. It will also be one of the largest randomised controlled trials of ACT for improving outcomes in long-term conditions. If the intervention proves to be efficacious, this treatment delivery could facilitate participation for those individuals whose impaired mobility prevents travelling to face-to-face sessions. As the main barrier to the adoption of any psychological intervention is the paucity of psychological services, the ACT intervention has been designed to be a self-guided programme requiring minimal support from highly qualified professionals.

Abbreviations:

ACT: Acceptance and Commitment Therapy, MD: Muscle Disease, SMC: Standard Medical Care QoL: Quality of Life, HRA: Health Research Authority, REC: Research Ethics Committee, AE: Adverse Event, SAE: Serious Adverse Event, SAR: Serious Adverse Reaction, SUSAR: Suspected Unexpected Serious Adverse Reaction, TSC-DMEC: Trial Steering Committee / Data Monitoring & Ethics Committee, PPI: patient and public involvement.

Acknowledgements: We are grateful to Daniel Thomas (PPI representative) and MD-UK representatives who commented on several drafts of the study materials at different stages. We are also grateful to our physiotherapy collaborators (Ms Joanna Reffin, Ms Emily Jay, Ms Sunitha Narayan, Ms Jade Donnelly, Mr Leslie Richards, Ms Kelly Orr).

Contributorship statement: MR,CG, SN, LM, AR and TC obtained funding for the study. MR, SN, CV, VE, LM, CG, AR and TC contributed to the study protocol. MR and TC are responsible for overall supervision. All authors read, contributed towards and approved of the final manuscript.

Competing interests: None disclosed.

Funding: National Institute for Health Research (NIHR) Research for Patient Benefit grant (Reference number: PB-PG-061331085). Muscular Dystrophy UK have been partners from the commencement of the study and funded the trial therapist.

For peer review only - http://bmjopen.bmj.com/site/about/guidelines.xhtml

The trial is sponsored by King's College Hospital NHS Foundation Trust and co-sponsored by King's College London.

References

1. Muscular Dystrophy Campaign. Muscle Disease: the Impact – Incidence and Prevalence of Neuromuscular Conditions in the UK. 2010. Published http://www.musculardystrophyuk.org/information-for-professionals/nhs-commissioners/resources-for-nhs-commissioners/neuromuscular-condition-prevalence/.

2. Della Marca G, Frusciante R, Vollono C, Iannaccone E, Dittoni S, Losurdo A, et al. Pain and the Alpha-Sleep Anomaly: A Mechanism of Sleep Disruption in Facioscapulohumeral Muscular Dystrophy. Pain Medicine. 2013;14(4):487-97.

3. Merrison A, Hanna M. Muscle disease. Practical neurology. 2009;9(1):54-65.

4. Burns TM, Graham CD, Rose MR, Simmons Z. Quality of life and measures of quality of life in patients with neuromuscular disorders. Muscle Nerve. 2012;46(1):9-25.

5. Graham CD, Rose MR, Grunfeld EA, Kyle SD, Weinman J. A systematic review of quality of life in adults with muscle disease. J Neurol. 2011;258(9):1581-92.

6. Graham CD, Weinman J, Sadjadi R, Chalder T, Petty R, Hanna MG, et al. A multicentre postal survey investigating the contribution of illness perceptions, coping and optimism to quality of life and mood in adults with muscle disease. Clin Rehabil. 2014 May;28(5):508-19.

7. Sadjadi R, Rose MR, Muscle Study Group. What determines quality of life in inclusion body myositis? J Neurol Neurosurg Psychiatry. 2010 Oct;81(10):1164-6.

8. Rose MR, Sadjadi R, Weinman J, Akhtar T, Pandya S, Kissel JT, et al. Role of disease severity, illness perceptions, and mood on quality of life in muscle disease. Muscle Nerve. 2012;46(3):351-9.

9. Nätterlund B, Gunnarsson L, Ahlström G. Disability, coping and quality of life in individuals with muscular dystrophy: a prospective study over five years. Disabil Rehabil. 2000;22(17):776-85.

10. Ahlstrom G, Sjoden P. Coping with illness-related problems and quality of life in adult individuals with muscular dystrophy. J Psychosom Res. 1996;41(4):365-76.

11. Graham CD, Rose MR, Hankins M, Chalder T, Weinman J. Separating emotions from consequences in muscle disease: Comparing beneficial and unhelpful illness schemata to inform intervention development. J Psychosom Res. 2013;74(4):320-6.

12. Graham CD, Gouick J, Krahé C, Gillanders D. A systematic review of the use of Acceptance and Commitment Therapy (ACT) in chronic disease and long-term conditions. Clin Psychol Rev. 2016;46:46-58.

13. Engel GL. The need for a new medical model: a challenge for biomedicine. Holistic Med. 1989;4(1):37-53.

For peer review only - http://bmjopen.bmj.com/site/about/guidelines.xhtml

14. Voet N, Bleijenberg G, Hendriks J, de Groot I, Padberg G, van Engelen B, et al. Both aerobic exercise and cognitive-behavioral therapy reduce chronic fatigue in FSHD: an RCT. Neurology. 2014 Nov 18;83(21):1914-22.

15. Graham CD, Simmons Z, Stuart SR, Rose MR. The potential of psychological interventions to improve quality of life and mood in muscle disorders. Muscle Nerve. 2015;52(1):131-6.

16. Hayes SC, Strosahl KD, Wilson KG. Acceptance and commitment therapy: An experiential approach to behavior change. Guilford Press; 1999.

17. McCracken LM, Morley S. The psychological flexibility model: a basis for integration and progress in psychological approaches to chronic pain management. The Journal of Pain. 2014;15(3):221-34.

18. Graham CD, Gouick J, Ferreira N, Gillanders D. The influence of psychological flexibility on life satisfaction and mood in muscle disorders. Rehabilitation psychology. 2016;61(2):210.

19. A-Tjak JG, Davis ML, Morina N, Powers MB, Smits JA, Emmelkamp PM. A meta-analysis of the efficacy of acceptance and commitment therapy for clinically relevant mental and physical health problems. Psychother Psychosom. 2015;84(1):30-6.

20. Öst L. The efficacy of Acceptance and Commitment Therapy: an updated systematic review and meta-analysis. Behav Res Ther. 2014;61:105-21.

21. Hann KE, McCracken LM. A systematic review of randomized controlled trials of Acceptance and Commitment Therapy for adults with chronic pain: Outcome domains, design quality, and efficacy. Journal of Contextual Behavioral Science. 2014;3(4):217-27.

22. Feros DL, Lane L, Ciarrochi J, Blackledge JT. Acceptance and Commitment Therapy (ACT) for improving the lives of cancer patients: a preliminary study. Psycho-Oncology. 2013;22(2):459-64.

23. Gregg JA, Callaghan GM, Hayes SC, Glenn-Lawson JL. Improving diabetes self-management through acceptance, mindfulness, and values: a randomized controlled trial. J Consult Clin Psychol. 2007;75(2):336.

24. Lundgren T, Dahl J, Melin L, Kies B. Evaluation of acceptance and commitment therapy for drug refractory epilepsy: a randomized controlled trial in South Africa—a pilot study. Epilepsia. 2006;47(12):2173-9.

25. Charova E, Dorstyn D, Tully P, Mittag O. Web-based interventions for comorbid depression and chronic illness: a systematic review. J Telemed Telecare. 2015 Jun;21(4):189-201.

26. Beatty L, Lambert S. A systematic review of internet-based self-help therapeutic interventions to improve distress and disease-control among adults with chronic health conditions. Clin Psychol Rev. 2013;33(4):609-22.

27. Hudson JL, Moss-Morris R, Game D, Carroll A, McCrone P, Hotopf M, et al. Improving distress in dialysis (iDiD): a feasibility two-arm parallel randomised controlled trial of an online cognitive behavioural therapy intervention with and without therapist-led telephone support for psychological distress in patients undergoing haemodialysis. BMJ Open. 2016 Apr 12;6(4):e011286,2016-011286.

28. Graham CD, Chalder T, Rose MR, Gavriloff D, McCracken LM, Weinman J. A pilot case series of a brief acceptance and commitment therapy (ACT)-based guided self-help intervention for improving quality of life and mood in muscle disorders. the Cognitive Behaviour Therapist. 2017;10.

For peer review only - http://bmjopen.bmj.com/site/about/guidelines.xhtml

29. Rose M. 188th ENMC International Workshop: Inclusion Body Myositis, 2–4 December 2011, Naarden, The Netherlands. Neuromuscul Disord. 2013;23(12):1044-55.

30. Wong A, Nyenhuis D, Black SE, Law LS, Lo ES, Kwan PW, et al. Montreal Cognitive Assessment 5-minute protocol is a brief, valid, reliable, and feasible cognitive screen for telephone administration. Stroke. 2015 Apr;46(4):1059-64.

31. Harris R. ACT made simple: An easy-to-read primer on acceptance and commitment therapy. New Harbinger Publications; 2009.

32. Plumb JC, Vilardaga R. Assessing treatment integrity in acceptance and commitment therapy: Strategies and suggestions. International Journal of Behavioral Consultation and Therapy. 2010;6(3):263.

33. Mayhew A, Cano S, Scott E, Eagle M, Bushby K, Muntoni F. Moving towards meaningful measurement: Rasch analysis of the North Star Ambulatory Assessment in Duchenne muscular dystrophy. Developmental Medicine & Child Neurology. 2011;53(6):535-42.

34. Cuthbert SC, Goodheart GJ. On the reliability and validity of manual muscle testing: a literature review. Chiropractic & osteopathy. 2007;15(1):4.

35. Vincent KA, Carr AJ, Walburn J, Scott DL, Rose MR. Construction and validation of a quality of life questionnaire for neuromuscular disease (INQoL). Neurology. 2007 Mar 27;68(13):1051-7.

36. Mundt JC, Marks IM, Shear MK, Greist JH. The Work and Social Adjustment Scale: a simple measure of impairment in functioning. Br J Psychiatry. 2002 May;180:461-4.

37. Zigmond AS, Snaith RP. The hospital anxiety and depression scale. Acta Psychiatr Scand. 1983;67(6):361-70.

38. Bjelland I, Dahl AA, Haug TT, Neckelmann D. The validity of the Hospital Anxiety and Depression Scale: an updated literature review. J Psychosom Res. 2002;52(2):69-77.

39. Fries JF, Spitz PW, Young DY. The dimensions of health outcomes: the health assessment questionnaire, disability and pain scales. J Rheumatol. 1982 Sep-Oct;9(5):789-93.

40. Jackson C, Barohn R, Gronseth G, Pandya S, Herbelin L. Inclusion body myositis functional rating scale: a reliable and valid measure of disease severity. Muscle Nerve. 2008;37(4):473-6.

41. Ferguson L, Scheman J. Patient global impression of change scores within the context of a chronic pain rehabilitation program. The Journal of Pain. 2009;10(4):S73.

42. Hayes SC, Strosahl K, Wilson KG, Bissett RT. Measuring experiential avoidance: A preliminary test of a working model. The psychological record. 2004;54(4):553.

43. Brown KW, Ryan RM. Perils and promise in defining and measuring mindfulness: Observations from experience. Clinical Psychology: Science and Practice. 2004;11(3):242-8.

44. McCracken L, Chilcot J, Norton S. Further development in the assessment of psychological flexibility: A shortened Committed Action Questionnaire (CAQ-8). European Journal of Pain. 2015;19(5):677-85.

45. Braun V, Clarke V. Using thematic analysis in psychology. Qualitative research in psychology. 2006;3(2):77-101.

46. Norman GR, Sloan JA, Wyrwich KW. Is it simple or simplistic? Med Care. 2003 May;41(5):599-600.

For peer review only

For peer review only - http://bmjopen.bmj.com/site/about/guidelines.xhtml

47. White PD, Sharpe MC, Chalder T, DeCesare JC, Walwyn R. Protocol for the PACE trial: A randomised controlled trial of adaptive pacing, cognitive behaviour therapy, and graded exercise as supplements to standardised specialist medical care versus standardised specialist medical care alone for patients with the chronic fatigue syndrome/myalgic encephalomyelitis or encephalopathy. BMC neurology. 2007;7(1):1.

48. World Medical Association. Declaration of Helsinki. Ethical principles for medical research involving human subjects. http://www.wma.net/e/policy/b3.htm. 2008.

49. Dixon JR. The international conference on harmonization good clinical practice guideline. Quality Assurance: Good Practice, Regulation, and Law. 1999;6(2):65-74.

Figure 1 Consort Diagram for ACTMuS

For peer review only

For peer review only - http://bmjopen.bmj.com/site/about/guidelines.xhtml

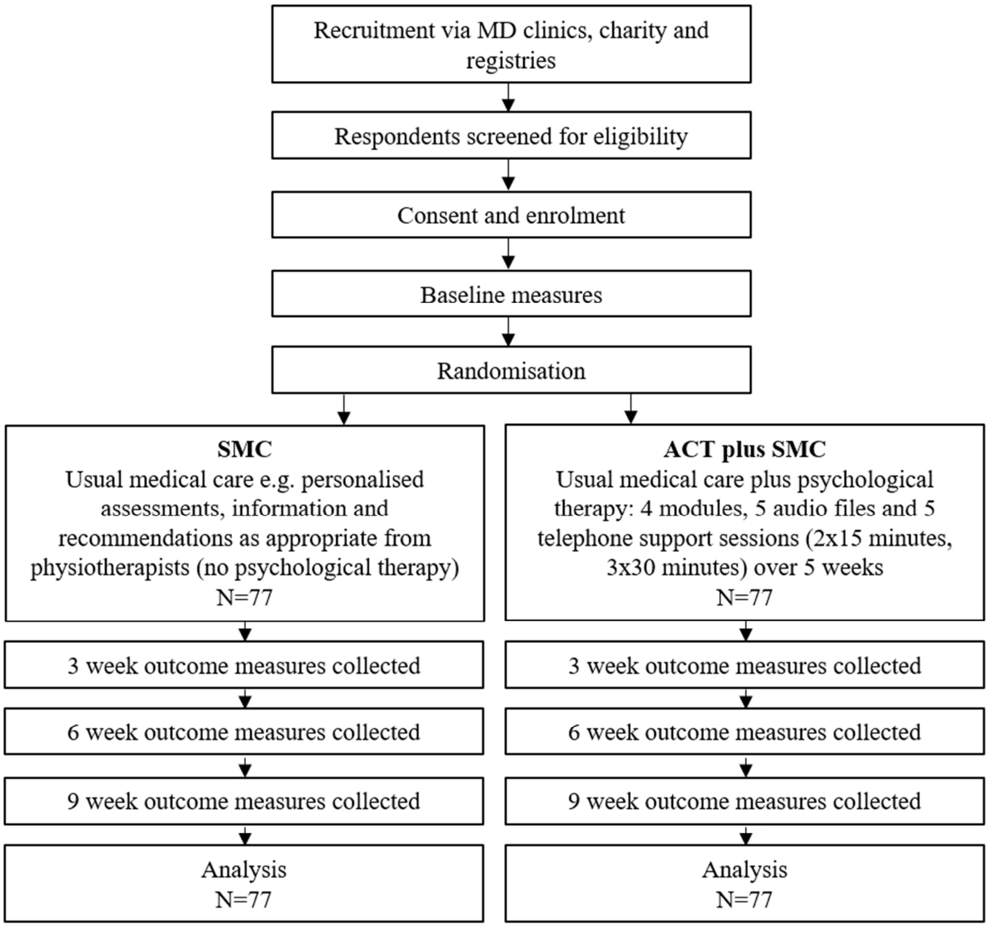
Recruitment via MD clinics, charity and registries
Respondents screened for eligibility
Consent and enrolment
Baseline measures
Randomisation
SMC
Usual medical care e.g. personalised assessments, information and recommendations as appropriate from physiotherapists (no psychological therapy)
N=77
ACT plus SMC
Usual medical care plus psychological therapy: 4 modules, 5 audio files and 5 telephone support sessions (2x15 minutes, 3x30 minutes) over 5 weeks
N=77
3 week outcome measures collected
3 week outcome measures collected
6 week outcome measures collected
6 week outcome measures collected
9 week outcome measures collected
9 week outcome measures collected
Analysis
N=77
Analysis
N=77

STANDARD PROTOCOL ITEMS: RECOMMENDATIONS FOR INTERVENTIONAL TRIALS

SPIRIT 2013 Checklist: Recommended items to address in a clinical trial protocol and related documents*

Section/item	Item No	Description	Addressed on page number
**Administrative information**			
Title	1	Descriptive title identifying the study design, population, interventions, and, if applicable, trial acronym	X (p.1)______
Trial registration	2a	Trial identifier and registry name. If not yet registered, name of intended registry	X(p.15)__________ _____
	2b	All items from the World Health Organization Trial Registration Data Set	_____N/A______
Protocol version	3	Date and version identifier	X (p. 2 Title Page_Authors_Administrative Information)_____
Funding	4	Sources and types of financial, material, and other support	X (p. 2 Title Page_Authors_Administrative Information)_____ ___________
Roles and responsibilities	5a	Names, affiliations, and roles of protocol contributors	X (p. 2 Title Page_Authors_Administrative Information)_____ _________

For peer review only

For peer review only - http://bmjopen.bmj.com/site/about/guidelines.xhtml

	5b	Name and contact information for the trial sponsor	_X ___________
	5c	Role of study sponsor and funders, if any, in study design; collection, management, analysis, and interpretation of data; writing of the report; and the decision to submit the report for publication, including whether they will have ultimate authority over any of these activities	_____________
	5d	Composition, roles, and responsibilities of the coordinating centre, steering committee, endpoint adjudication committee, data management team, and other individuals or groups overseeing the trial, if applicable (see Item 21a for data monitoring committee)	____________
**Introduction**			
Background and rationale	6a	Description of research question and justification for undertaking the trial, including summary of relevant studies (published and unpublished) examining benefits and harms for each intervention	_X (p. 4-5)____
	6b	Explanation for choice of comparators	_X (p. 4-5)___
Objectives	7	Specific objectives or hypotheses	_X (p.5)_____
Trial design	8	Description of trial design including type of trial (eg, parallel group, crossover, factorial, single group), allocation ratio, and framework (eg, superiority, equivalence, noninferiority, exploratory)	__X (p.5)__
**Methods: Participants, interventions, and outcomes**			
Study setting	9	Description of study settings (eg, community clinic, academic hospital) and list of countries where data will be collected. Reference to where list of study sites can be obtained	___X (p.6)__
Eligibility criteria	10	Inclusion and exclusion criteria for participants. If applicable, eligibility criteria for study centres and individuals who will perform the interventions (eg, surgeons, psychotherapists)	__X (p.6)__
Interventions	11a	Interventions for each group with sufficient detail to allow replication, including how and when they will be administered	__X (p.6-8)__

For peer review only - http://bmjopen.bmj.com/site/about/guidelines.xhtml

	11b	Criteria for discontinuing or modifying allocated interventions for a given trial participant (eg, drug dose change in response to harms, participant request, or improving/worsening disease)	____________
	11c	Strategies to improve adherence to intervention protocols, and any procedures for monitoring adherence (eg, drug tablet return, laboratory tests)	______X (p.7)__
	11d	Relevant concomitant care and interventions that are permitted or prohibited during the trial	____X (p.6)__
Outcomes	12	Primary, secondary, and other outcomes, including the specific measurement variable (eg, systolic blood pressure), analysis metric (eg, change from baseline, final value, time to event), method of aggregation (eg, median, proportion), and time point for each outcome. Explanation of the clinical relevance of chosen efficacy and harm outcomes is strongly recommended	___X (p.8-10)_
Participant timeline	13	Time schedule of enrolment, interventions (including any run-ins and washouts), assessments, and visits for participants. A schematic diagram is highly recommended (see Figure)	___X (p. 11-12)_
Sample size	14	Estimated number of participants needed to achieve study objectives and how it was determined, including clinical and statistical assumptions supporting any sample size calculations	__X (p.13)__
Recruitment	15	Strategies for achieving adequate participant enrolment to reach target sample size	__X (p.13)______

Methods: Assignment of interventions (for controlled trials)

Allocation:

Sequence generation	16a	Method of generating the allocation sequence (eg, computer-generated random numbers), and list of any factors for stratification. To reduce predictability of a random sequence, details of any planned restriction (eg, blocking) should be provided in a separate document that is unavailable to those who enrol participants or assign interventions	__X (p.13)_____
Allocation concealment mechanism	16b	Mechanism of implementing the allocation sequence (eg, central telephone; sequentially numbered, opaque, sealed envelopes), describing any steps to conceal the sequence until interventions are assigned	___X (p.13)___
Implementation	16c	Who will generate the allocation sequence, who will enrol participants, and who will assign participants to interventions	____X (p.13)____

Blinding (masking)	17a	Who will be blinded after assignment to interventions (eg, trial participants, care providers, outcome assessors, data analysts), and how	____X (p.13)____
	17b	If blinded, circumstances under which unblinding is permissible, and procedure for revealing a participant's allocated intervention during the trial	_____X (p.13)___
**Methods: Data collection, management, and analysis**			
Data collection methods	18a	Plans for assessment and collection of outcome, baseline, and other trial data, including any related processes to promote data quality (eg, duplicate measurements, training of assessors) and a description of study instruments (eg, questionnaires, laboratory tests) along with their reliability and validity, if known. Reference to where data collection forms can be found, if not in the protocol	___X (p.13)___
	18b	Plans to promote participant retention and complete follow-up, including list of any outcome data to be collected for participants who discontinue or deviate from intervention protocols	____X (p.13)___
Data management	19	Plans for data entry, coding, security, and storage, including any related processes to promote data quality (eg, double data entry; range checks for data values). Reference to where details of data management procedures can be found, if not in the protocol	____X (p.14)___
Statistical methods	20a	Statistical methods for analysing primary and secondary outcomes. Reference to where other details of the statistical analysis plan can be found, if not in the protocol	___X (p.14)___
	20b	Methods for any additional analyses (eg, subgroup and adjusted analyses)	_____ ___
	20c	Definition of analysis population relating to protocol non-adherence (eg, as randomised analysis), and any statistical methods to handle missing data (eg, multiple imputation)	___X (p.14)____
**Methods: Monitoring**			
Data monitoring	21a	Composition of data monitoring committee (DMC); summary of its role and reporting structure; statement of whether it is independent from the sponsor and competing interests; and reference to where further details about its charter can be found, if not in the protocol. Alternatively, an explanation of why a DMC is not needed	_____________
	21b	Description of any interim analyses and stopping guidelines, including who will have access to these interim results and make the final decision to terminate the trial	_____________

Harms	22	Plans for collecting, assessing, reporting, and managing solicited and spontaneously reported adverse events and other unintended effects of trial interventions or trial conduct	__X (p. 14-15)__
Auditing	23	Frequency and procedures for auditing trial conduct, if any, and whether the process will be independent from investigators and the sponsor	_____________
**Ethics and dissemination**			
Research ethics approval	24	Plans for seeking research ethics committee/institutional review board (REC/IRB) approval	___X (p. 15)__
Protocol amendments	25	Plans for communicating important protocol modifications (eg, changes to eligibility criteria, outcomes, analyses) to relevant parties (eg, investigators, REC/IRBs, trial participants, trial registries, journals, regulators)	_____________
Consent or assent	26a	Who will obtain informed consent or assent from potential trial participants or authorised surrogates, and how (see Item 32)	___X (p.15)__
	26b	Additional consent provisions for collection and use of participant data and biological specimens in ancillary studies, if applicable	_____________
Confidentiality	27	How personal information about potential and enrolled participants will be collected, shared, and maintained in order to protect confidentiality before, during, and after the trial	______X (p.14)__
Declaration of interests	28	Financial and other competing interests for principal investigators for the overall trial and each study site	__X (p.15)____
Access to data	29	Statement of who will have access to the final trial dataset, and disclosure of contractual agreements that limit such access for investigators	____________
Ancillary and post-trial care	30	Provisions, if any, for ancillary and post-trial care, and for compensation to those who suffer harm from trial participation	____________
Dissemination policy	31a	Plans for investigators and sponsor to communicate trial results to participants, healthcare professionals, the public, and other relevant groups (eg, via publication, reporting in results databases, or other data sharing arrangements), including any publication restrictions	____X (p.15)_
	31b	Authorship eligibility guidelines and any intended use of professional writers	____________

For peer review only

	31c	Plans, if any, for granting public access to the full protocol, participant-level dataset, and statistical code	____________
**Appendices**			
Informed consent materials	32	Model consent form and other related documentation given to participants and authorised surrogates	______________
Biological specimens	33	Plans for collection, laboratory evaluation, and storage of biological specimens for genetic or molecular analysis in the current trial and for future use in ancillary studies, if applicable	_____________

*It is strongly recommended that this checklist be read in conjunction with the SPIRIT 2013 Explanation & Elaboration for important clarification on the items. Amendments to the protocol should be tracked and dated. The SPIRIT checklist is copyrighted by the SPIRIT Group under the Creative Commons "Attribution-NonCommercial-NoDerivs 3.0 Unported" license.

For peer review only

For peer review only - http://bmjopen.bmj.com/site/about/guidelines.xhtml
